# CARAT: A novel method for allelic detection of DNA copy number changes using high density oligonucleotide arrays

**DOI:** 10.1186/1471-2105-7-83

**Published:** 2006-02-21

**Authors:** Jing Huang, Wen Wei, Joyce Chen, Jane Zhang, Guoying Liu, Xiaojun Di, Rui Mei, Shumpei Ishikawa, Hiroyuki Aburatani, Keith W Jones, Michael H Shapero

**Affiliations:** 1 Affymetrix, Inc. 3420 Central Expressway, Santa Clara CA 95051, USA; 2 University of Tokyo, Genome Science Division Research Center for Advanced Science and Technology, 4-6-1 Komaba, Meguro, 153-8904, Tokyo

## Abstract

**Background:**

DNA copy number alterations are one of the main characteristics of the cancer cell karyotype and can contribute to the complex phenotype of these cells. These alterations can lead to gains in cellular oncogenes as well as losses in tumor suppressor genes and can span small intervals as well as involve entire chromosomes. The ability to accurately detect these changes is central to understanding how they impact the biology of the cell.

**Results:**

We describe a novel algorithm called CARAT (Copy Number Analysis with Regression And Tree) that uses probe intensity information to infer copy number in an allele-specific manner from high density DNA oligonuceotide arrays designed to genotype over 100, 000 SNPs. Total and allele-specific copy number estimations using CARAT are independently evaluated for a subset of SNPs using quantitative PCR and allelic TaqMan reactions with several human breast cancer cell lines. The sensitivity and specificity of the algorithm are characterized using DNA samples containing differing numbers of X chromosomes as well as a test set of normal individuals. Results from the algorithm show a high degree of agreement with results from independent verification methods.

**Conclusion:**

Overall, CARAT automatically detects regions with copy number variations and assigns a significance score to each alteration as well as generating allele-specific output. When coupled with SNP genotype calls from the same array, CARAT provides additional detail into the structure of genome wide alterations that can contribute to allelic imbalance.

## Background

The cancer cell karyotype is often complex and can include a range of molecular alterations that span mutations at the single nucleotide level to extensive rearrangements involving whole chromosomes. The activation of oncogenes as the result of DNA amplifications and the inactivation of tumor suppressor genes as the result of DNA deletions can both contribute to the cancer cell phenotype. With the recent identification of large scale copy number polymorphisms (CNPs) in the human genome as well, it is increasingly clear that a detailed understanding of the role of genomic alterations and structure will be important in the context of both the normal and disease state [1-8]. Over the years many experimental approaches have been described that have increased our knowledge of the cancer genome. These include genome-wide approaches such as array comparative genomic hybridization (array CGH) to cDNA clones [9,10], bacterial artificial chromosomes (BACs), P1 artificial chromosomes (PACs) [11,12], and long oligonucleotides [13-15], restriction landmark genome scanning (RLGS) [16], spectral karyotyping (SKY) [17], molecular subtraction such as RDA [18], digital karyotyping [19,20], and end sequence profiling (ESP) [21] as well as more focused approaches such as high-throughput quantitative PCR (QPCR) and fluorescence *in situ *hybridization (FISH) [22]. While no single experimental approach allows the comprehensive analysis of all types of chromosomal aberrations, array-based approaches offer the greatest potential for high resolution genome-wide scans.

High density DNA oligonucleotide arrays using light-directed parallel chemical synthesis allow unprecedented levels of genetic information to be captured in single experiments [23-25]. The completion of the human genome sequence, coupled with the emergence of single nucleotide polymorphisms (SNPs) as the most common form of genetic variation among individuals, has led to a variety of applications for high density genotyping arrays. In the past, these arrays have been used in traditional loss of heterozygosity (LOH) analysis using standard approaches of multiplex PCR for DNA target generation [26-28]. More recently, a DNA target generation method using complexity reduction by single primer PCR, termed whole genome sampling assay (WGSA), was developed for simultaneous genotyping of over 10, 000 SNPs on a single array [29,30]. This array has been used for hierarchical tumor clustering based on LOH patterns with human lung cancer cell lines [31], the characterization of LOH progression in samples from children with acute lymphoblastic leukemia who relapse after chemotherapy [32], and for a case-control study of esophageal squamous cell carcinoma (ESCC) [33]. Furthermore, the array has also been shown to accurately detect genome-wide DNA copy number changes [34-36]. By coupling SNP genotypes with copy number information, detailed insight into genomic structure can be gleaned. For example, genomic regions displaying LOH can be differentiated into regions with hemizygous deletions and regions with no change in copy number, i.e. copy neutral events, and genomic regions undergoing copy number loss without LOH can also be detected [34,37,38]. Allelic imbalance, of which LOH is one example, can also occur when one allele is preferentially amplified relative to the other allele. The coupling of genotypic information with copy number information from a single array allows genome-wide allele-typing to be carried out [37,39,40]. This type of combined analysis can not be made using approaches such as array CGH (reviewed in [41]) and thus underscores the potential power of identifying novel genomic alterations using high density SNP genotyping arrays.

Recently, the WGSA assay has been extended to allow highly accurate SNP genotyping of over 100,000 SNPs from two arrays [42]. With an average inter-marker distance of 23.6 kb, the arrays provide dense enough coverage to enable whole-genome association studies [43]. In this report we describe a novel algorithm termed CARAT (Copy Number Analysis with Regression And Tree) that uses probe intensity information from the GeneChip^® ^Mapping 100 K set for genome-wide allele-specific copy number estimations. CARAT is predicated on the use of the highly accurate genotypes derived from the array to evaluate allelic dose responses on a SNP-by-SNP basis, thereby allowing the copy number output for each allele to be determined. We show using DNA samples from established cell lines that different types of genetic alterations (amplifications, deletions, and LOH) are readily detectable using an allele-specific copy number approach. Thus the coupling of SNP genotypes with allele-specific copy number information may provide new insight into complex genomic alterations, such as regions undergoing allelic imbalance due to differential allelic amplification.

## Results and discussion

We have previously described the use of the 10 K SNP genotyping array for chromosomal copy number analysis [34,35]. Recently, the ability to genotype 100 K SNPs on a set of arrays has become available and these arrays have been used for high resolution copy number analysis [44]. As with the 10 K array, the 100 K array set uses the WGSA target preparation scheme in which single primer PCR amplification of specific fractions of the genome is carried out. The primary difference with the 100 K WGSA method is the use of two separate restriction enzymes that each generates a higher complexity fraction estimated to be ~300 Mb. In this report we describe a new algorithm called CARAT. In CARAT, a complex normalization scheme that incorporates both restriction fragment and probe sequence information is applied on individual arrays to reduce any systematic error and to increase comparability across experiments. Probes for each SNP are tested for the ability to support an allelic dosage response using a set of normal individuals in which the 'AA', 'AB', and 'BB' genotypes intrinsically represent zero, one, and two copies of the 'B' allele and two, one, and zero copies of the 'A' allele. Probes displaying a strong dosage response are employed in a regression framework to estimate allele-specific copy number. For any target sample, the sum of the copy number estimates from the two alleles is compared against the reference set to derive a significance measure of the deviation from the diploid state. Smoothing is used on the estimated copy number and its corresponding significance to further reduce the experimental and technical noise. Regression trees [45] are applied on the smoothed result to partition the genome into regions with different copy numbers and to assign an overall significance to such changes.

WGSA 100 K arrays perform robustly for SNP genotyping, with call rates, reproducibility, and accuracy greater than 99%, 99.7%, and 99.7% respectively [42]. Since CARAT does rely on genotype calls, any SNPs with systematic errors in the calls could potentially bias the results. In order to prevent any such bias, only genotypes with stringent confidence rank scores are used, and SNPs that do not meet this criterion are scored as "no calls". Although the majority of steps in CARAT do not make use of "no call" SNPs, there are several steps that do use them, in which case they are always compared against all genotypes to reduce any systematic bias in the analysis.

Among the full complement of over 116 K SNPs, 91,908 (79.1%) display a high allelic dose response as defined by a linear correlation greater than 0.8 between the target concentration and chip intensity. Among these SNPs, 51097 (55.6%) incorporated information from all 20 perfect match (PM) probes (10 PM 'A' allele (PMa) and 10 PM 'B' allele (PMb)), 31857 (34.6%) incorporated information from 15 ~ 19 probes, 8268 (8.9%) incorporated information from 10 ~ 14 probes, 682 (0.74%) incorporated information from 5 ~ 9 probes, 4 (0.004%) incorporated information from 3 or 4 out of 20 probes, and no SNPs used less than 3 probes. This subset of 91, 908 SNPs, with an average inter-marker distance of 30.5 kb, was used in CARAT for copy number estimations.

The performance of CARAT was evaluated with a set of test samples that included 90 normal individuals, DNA samples with varying numbers of X chromosomes (1X to 5X), and several human breast cancer cell lines that harbor both low level and high level copy number alterations. None of these test samples have any overlap with the 128 training samples that are used to establish and tune the CARAT models.

**Figure 1:**
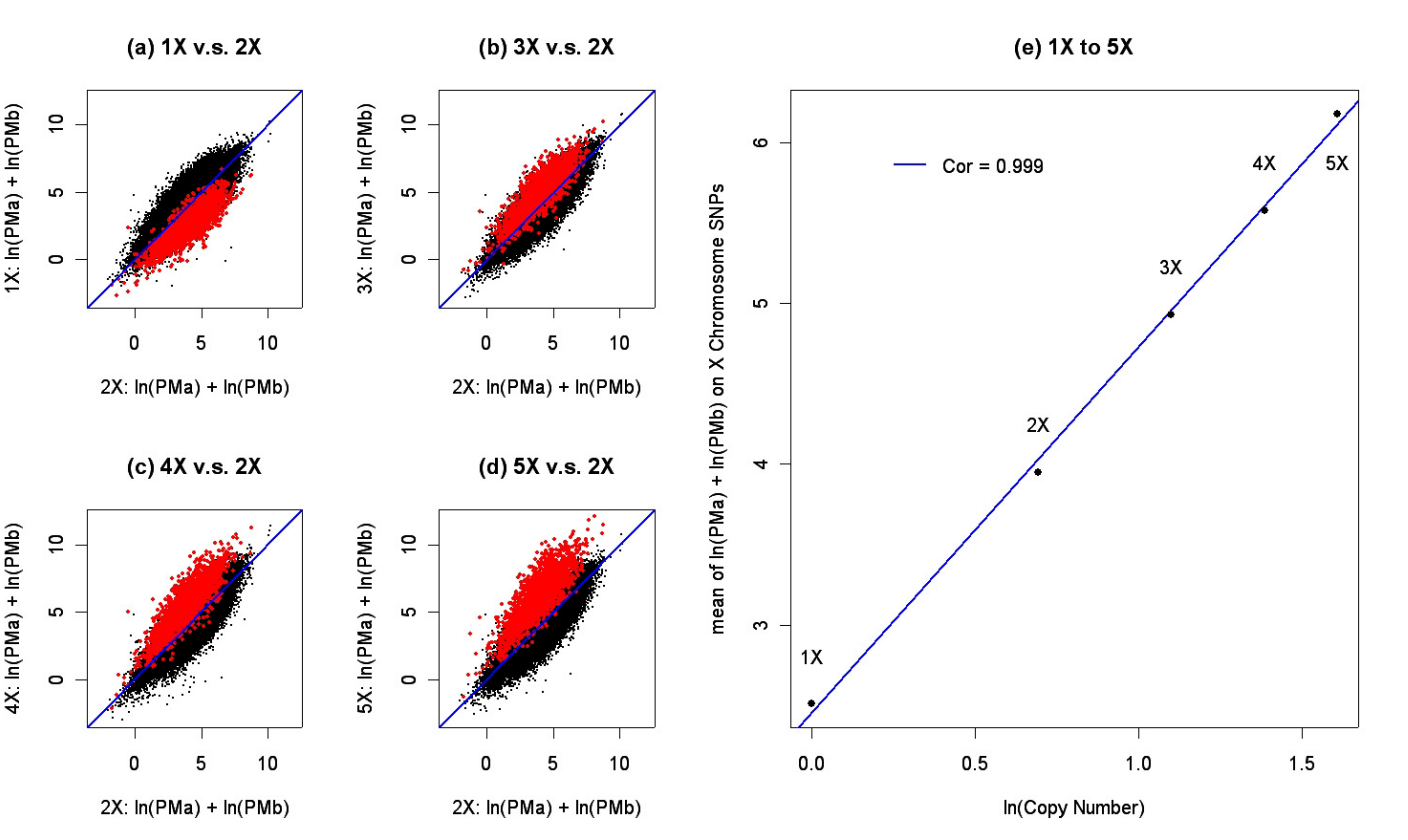
Panels a-d show the standardized ln(PMa) + ln(PMb) intensity for the 1X, 3X, 4X, and 5X DNA samples relative to the intensity of the 2X DNA sample. Black data points correspond to autosomal SNPs and red data points correspond to the 1,955 X-chromosome SNPs. The blue line in each panel represents the Y = X line. Panel e shows the relationship between the natural log-transformed copy number and the natural log-transformed intensity. The x-axis is the natural log-transformed copy number and the y axis is the average ln(PMa) + ln(PMb) intensity across 1,955 SNPs. The blue line is the regression using the average intensity as the response and the natural log-transformed copy number as the predictor.

The relationship between DNA copy number and fluorescent intensity of the SNP hybridization signal was evaluated using genomic DNA derived from cell lines with a defined number of X chromosomes (1X to 5X). Among the 91,908 selected SNPs, 1,955 map to the X chromosome. A normal 2X (NA15029) female sample was used as the reference for comparisons to the 1X, 3X, 4X, and 5X samples. The results are summarized in Figure 1. Panels a-d show that there is a high linear correlation among the sample pairs, and only X-chromosome SNPs (labeled in red) show intensity profile shifts across the four panels while the autosomal SNPs (labeled in black) remain static. Panel e indicates that there is a strong linear relationship between the log transformed copy number and the log transformed intensity. These results show that the 100 K WGSA PCR fractions maintain a nice dose response between the input template copy number and the post hybridization SNP fluorescent intensity.

Table 1 summarizes the true positive rates for detection of X chromosome changes using the 1X, 3X, 4X and 5X DNA samples along with the false positive rate of detection of autosomal SNPs deviating from the diploid state using the test set of 90 normal individuals. Values are computed for all samples at several different stages of CARAT and at various significance thresholds. The results indicate that the addition of the kernel smoothing and the tree partitioning steps improves the true positive rate and decreases the false positive rate; only at the most stringent significance cut-off does the false positive rate exceed the expected value. Moreover, with the regression tree partition function, CARAT defines the alterations on the X chromosome as a single region for all four samples with a very high significance. The overall copy number estimates (and significance) for the X chromosome using the 1X to 5X samples are: 1X:0.92 (1.99 × 10^-4^), 3X:3.21 (8.13 × 10^-6^), 4X:4.36 (6.15 × 10^-12^) and 5X:5.74 (1.50 × 10^-16^).

**Table 1:** Estimation of true positive and false positive rates under varying significance thresholds using 1X to 5X samples and 90 normal test samples.

Sample and Data	Stage	p-value < 10^-2^		p-value < 10^-4^		p-value < 10^-6^		Total
		
		Count	Percent	Count	Percent	Count	Percent	Count
90 normal samples (autosomes)	SP	409049	5.05%	42182	0.52%	11154	0.14%	8095770
	KS	40247	0.497%	3302	0.041%	770	0.009%	8095770
	TR	5417	0.067%	506	0.006%	167	0.002%	8095770
1X	SP	1441	73.71%	694	35.50%	286	14.63%	1955
	KS	1780	91.05%	714	36.52%	111	5.68%	1955
	TR	p-value = 1.99 × 10^-4^						1955
3X	SP	1271	65.01%	879	44.96%	603	30.84%	1955
	KS	1707	87.31%	1136	58.11%	547	27.98%	1955
	TR	p-value = 8.13 × 10^-6^						1955
4X	SP	1726	88.29%	1523	77.90%	1343	68.70%	1955
	KS	1929	98.67%	1861	95.19%	1697	86.80%	1955
	TR	p-value = 6.15 × 10^-12^						1955
5X	SP	1884	96.37%	1801	92.12%	1724	88.18%	1955
	KS	1950	99.74%	1933	98.87%	1907	97.54%	1955
	TR	p-value = 1.50 × 10^-16^						1955

Notes:

^1 ^SP: Single point analysis

^2 ^KS: Kernel smoothing with 100 kb window after single point analysis

^3 ^TR: Tree partitioning of the genome on the kernel smoothed result.

**Figure 2:**
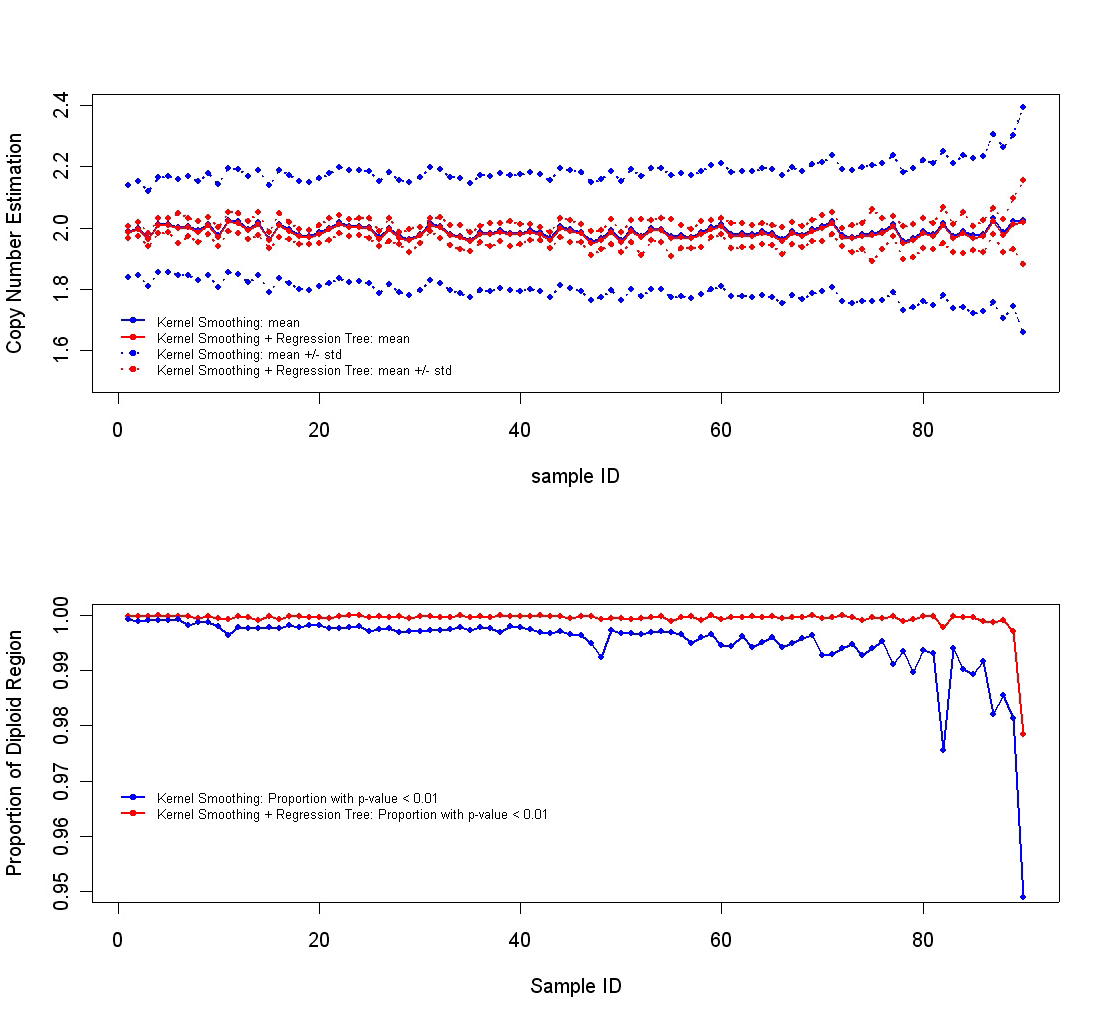
The upper panel shows the mean autosomal SNP copy number and the associated standard deviation using kernel smoothing alone and kernel smoothing combined with the tree partition for each of the 90 normal samples in the independent test set. The solid lines correspond to the mean estimation and the dotted lines represent the mean plus or minus one standard deviation. The lower panel shows the proportion of the genome (autosomal chromosomes only) that is determined to be in the normal diploid state for the 90 individuals. The blue colored lines in both panels represent results using kernel smoothing alone while the red colored lines represent results from kernel smoothing combined with the regression tree partition.

These 90 HapMap CEPH samples (30 trios) thus served as an independent test set to evaluate the accuracy of the SNP copy number estimations as well as the algorithm's false-positive rate (FPR). These samples were assumed to represent normal diploid genomes which do not harbor extensive genomic deletions or amplifications. Although these samples could contain copy number polymorphisms, they are relatively rare and were not considered in this analysis, which potentially could lead to an overestimation of the true false positive rate. There were 89,953 autosomal SNPs among the total of 91,908 selected SNPs that were examined across the 90 individuals for a total of 8,095,770 data points; X chromosome SNPs were excluded due to copy number differences between males and females. One possible explanation for the higher-than-expected false positive rate at the stringent p-value of less than 10^-6 ^is that they are not false positives but rather true and significant copy number polymorphisms occurring in normal people. For example, there were 167 data points identified with a significance level less than 10^-6^. Among them, 72 SNPs were derived from a common amplified region on chromosome 8 from two samples originating from the same trio, namely NA12802 (child) and NA12814 (father), with each sample showing the same 36 significant SNPs. Although this amplified region (~16.34–16.85 Mb) has not been independently verified using QPCR, it does partially overlap with a BAC clone (RP11-90I3) from 8p22 that has detected a CNP [2] and thus may represent a CNP that is transmitted through generations. The copy number estimation of each autosomal SNP across these 90 test samples also has relatively low variation as shown in the upper panel of Figure 2. The mean copy number estimate across all autosomal SNPs ranges from 1.951 to 2.032 and is similar whether using only kernel smoothing or kernel smoothing combined with regression trees. However, by adding the regression tree as the final partition step, the standard deviation is dramatically reduced by an average of 81.4%, and the range changes from (0.149, 0.367) to (0.019, 0.037). The lower panel shows the proportion of the genome on a per-sample basis that does not contain any significant changes. Using regression trees, there are many more regions identified as diploid as compared to using kernel smoothing only, indicating an improvement in the apparent false positive rate.

**Figure 3:**
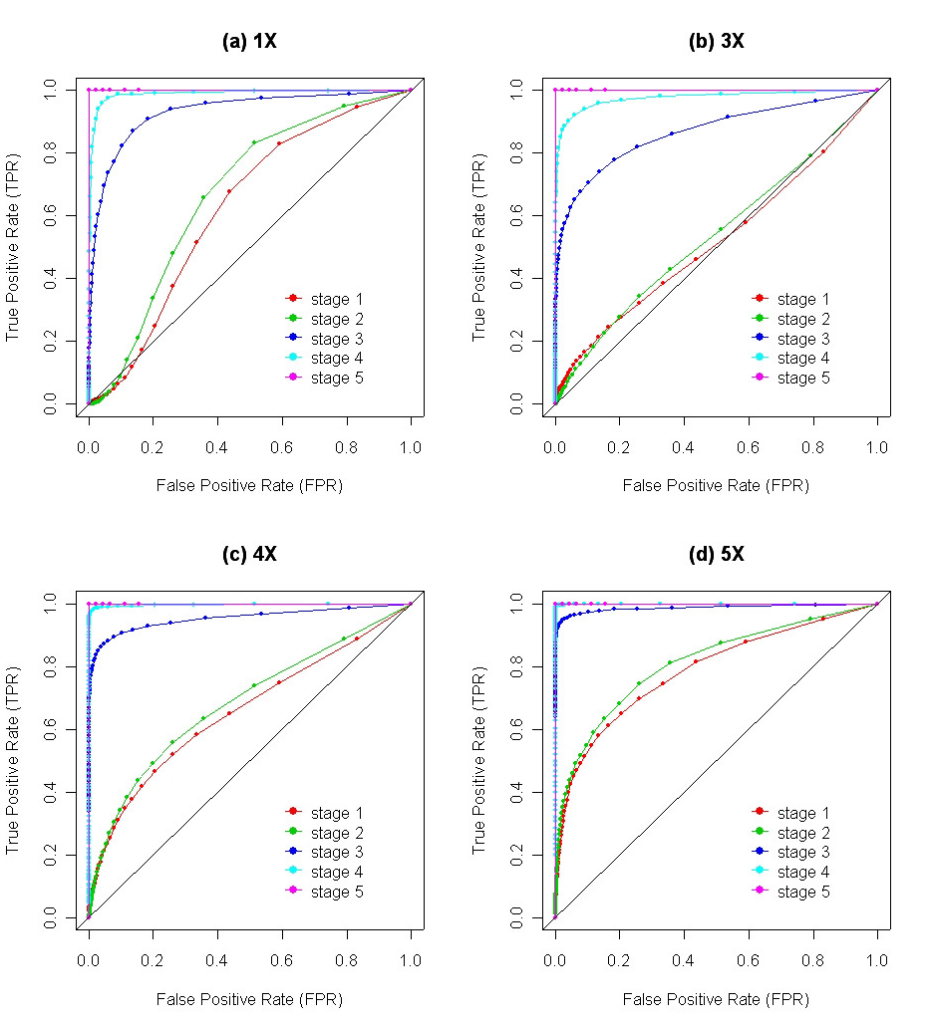
Each panel shows a series of ROC curves derived from different stages of CARAT using samples with X chromosome alterations. Stage 1: Single point analysis that contains no probe selection, no intensity adjustment on fragment length and GC content; and no intensity adjustment on the reference mean. Stage 2: Stage 1 plus probe selection. Stage 3: Stage 2 plus intensity adjustment on the fragment length and GC content and intensity adjustment on the reference mean. Stage 4: Stage 3 plus kernel smoothing with a 100 kb window. Stage 5: Stage 4 plus genome partitioning using the regression tree. This figure should be viewed in conjunction with Table 2 which summarizes the area under the ROC curves.

Receiver Operating Characteristic (ROC) curves were used to evaluate the overall sensitivity (true positive fraction) and specificity (true negative fraction) of different stages of CARAT. The curves are calculated using 1,955 X chromosome SNPs, with the false positive rate estimated by averaging the individual false positive signals across the 47 female samples present in the total set of 90 normal individuals. Figure 3 shows the ROC curves derived from different stages of the algorithm using DNA samples with differing numbers of X-chromosomes; Table 2 summarizes the area under those curves depicted in Figure 3. The most significant improvement comes from the adjustment based on fragment length, GC content and reference mean; the AUC (Area Under the Curve) increases about 50% for the 1X, 3X and 4X samples and 21.5% for the 5X sample. The improvement from probe-selection is relatively modest, resulting in an overall increase across the samples of about 5%. Although adding kernel smoothing in stage 4 and tree partitions in stage 5 does not substantially increase the AUC, these steps are nevertheless critical. These two steps drive the AUC towards 1, ensuring high sensitivity while keeping the specificity extremely low, which is a necessity since nearly 92 K SNPs are simultaneously being examined. In the tree partitioning step (stage 5), the ROC curves are ideal for the 4X and the 5X samples, rendering an AUC of 1. For the 1X and the 3X samples, the ROC curves are not smooth but rather step-functions that achieve a 100% true positive rate with a minimum false positive rate. This occurs because for each case the regression tree step successfully identifies the variations on the X chromosome as one altered region and assigns the region a high significance score that rarely occurs in normal female samples.

**Table 2:** Area under the ROC curve derived from different stages of the CARAT method.

Area under ROC Curve	1X	3X	4X	5X
Stage 1	0.6240	0.5188	0.6575	0.7863
Stage 2	0.6732	0.5384	0.6840	0.8128
Stage 3	0.9319	0.8987	0.9546	0.9877
Stage 4	0.9887	0.9759	0.9974	0.9995
Stage 5	0.9999	0.9999	1.0000	1.0000

Note:

^1^The specificity is estimated using the 47 female samples that are a subset of the 90 normal test set samples and is restricted to the 1955 X chromosome SNPs. The sensitivity is estimated on 1955 X chromosome SNPs using DNA samples with 1X, 3X, 4X and 5X chromosomes.

^2^Stage 1: Single point analysis: no probe selection, no intensity adjustment on fragment length and GC content; no intensity adjustment on reference mean.

^3^Stage 2: Stage 1 plus probe selection.

^4^Stage 3: Stage 2 plus intensity adjustment on the fragment length and GC content and intensity adjustment on the reference mean.

^5^Stage 4: Stage 3 plus kernel smoothing with a 100 kb window.

^6^Stage 5: Stage 4 plus genome partitioning using the regression tree.

The previous DNA samples with variable X chromosome content provided a means to evaluate the algorithm using large alterations that span the length of an entire chromosome. In order to better evaluate the performance of CARAT when the alterations were of low level copy number changes that did not span entire chromosomes, as well as evaluating CARAT relative to other methods, a series of experiments were carried out. These experiments included QPCR on 69 SNPs chosen from the cancer cell line SK-BR-3, QPCR around the HER2/neu region using three cancer cell lines; and allele-specific TaqMan on nine SNPs across two cell lines coupled with DNA sequence analysis. All experimental results show a high correlation with CARAT-derived estimates, indicating that the algorithm in combination with the Mapping 100 K array set can detect chromosomal copy number changes in an accurate and quantitative manner.

**Figure 4:**
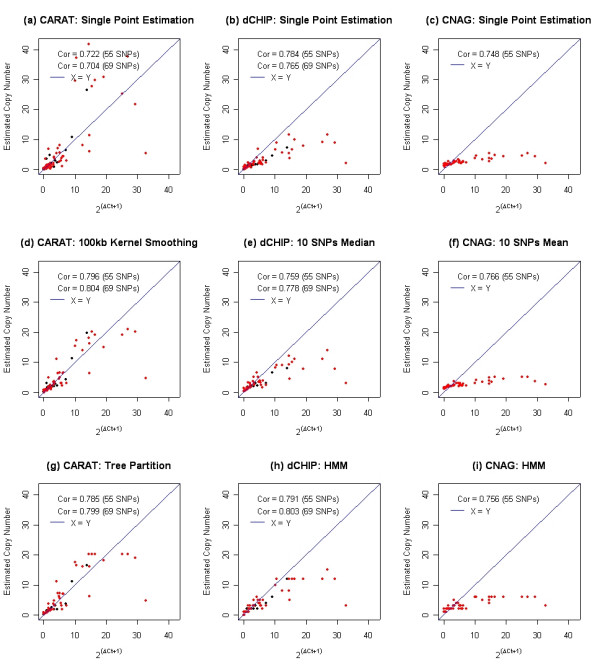
These nine panels show comparisons among CARAT, dCHIP and CNAG qPCR results of 69 autosomal SNPs from the human breast cancer cell line SK-BR-3. In each scatter plot the x-axis is the copy number derived from QPCR and the y-axis is the copy number derived from one of the three algorithms. ΔCt denotes the difference between the normal DNA sample versus SK-BR-3. The threshold cycle (Ct) is the cycle number at which the reporter fluorescence passes a fixed threshold above baseline. A positive ΔCt suggests an amplification while a negative ΔCt suggests a deletion. The copy number of SK-BR-3 based on QPCR is inferred as 2^(ΔCt + 1)^. The red points are the 55 SNPs that were included in the CNAG analysis; the black points are the 14 additional SNPs that were included in dCHIP and CARAT analysis but were excluded from CNAG. Correlations are calculated for each of these two different SNP sets. The blue line in each panel represents the Y = X line. Panels (a), (b), and (c) compare single point analysis across the three methods; panels (d), (e), and (f) compare smoothing across neighboring points; panels (g), (h), and (i) compare genome partitioning across the three methods.

We used QPCR as an independent method to determine the total copy number of 69 autosomal SNPs from SK-BR-3. These results were then compared to copy number output from CARAT and two additional algorithms used for Mapping 100 K copy number analysis, namely dCHIP [46] and CNAG [47]. These SNPs are derived from regions of SK-BR-3 that display copy number gains and losses as well as regions with no detectable changes, covering 16 of the 22 autosomes and more than 60 different regions; 10 of the 69 SNPs have a copy number between 1.5 and 2.5, indicating no major alterations from diploidy; 14 of the 69 SNPs have been excluded from CNAG because the SNPs reside on restriction fragments shorter than 500 bp and are resistant to the compensations used in CNAG (Table 3). Figure 4 summarizes the comparison of the correlations between the copy number derived from the three algorithms at different stages and the copy number derived from QPCR. The results show that the correlation values across the three methods are not significantly different. However, both CNAG and dCHIP under-estimate the total DNA copy number, although to different extents. In CNAG, neither the averaging across neighboring points nor the HMM procedure leads to a significant increase in the correlation. In contrast, the HMM step in dCHIP and the kernel smoothing step in CARAT do improve their respective correlations. In Table 4, the performance of the three methods is examined by evaluating the sensitivity and specificity using the same 69 QPCR results. In CNAG, because the estimation is biased towards the normal diploid state, it achieves perfect specificity while demonstrating substantially lower sensitivity compared to the other two methods. Although dCHIP and CARAT have similar performances with one another, CARAT has a higher sensitivity in the single-point estimation step and the smoothing step while dCHIP has higher specificity in the smoothing step. In dCHIP, the averaging and HMM steps steadily improve both the sensitivity and the specificity while in CARAT the sensitivity remains the same while the specificity is substantially increased through the three stages. Neither dCHIP nor CNAG has a significance measure associated with the estimated copy number at the single SNP level. Thus, in an attempt to compare the three algorithms, only copy number output from CARAT has been used, rather than the combination of copy number output and the associated p-values. The only exception to this is the analysis of the tree partitioning step in which algorithm-based true negatives are defined as SNPs with p-value > 0.005 and algorithm-based true positives are defined as SNPs with p-value < 0.005. Although the p-values from the regression tree step may not have a direct probabilistic interpretation, they nonetheless are derived from individual p-value estimates and thus serve as confidence scores that measures how significantly the region deviates from the diploid state. The use of a significance level rather than a copy number value as a threshold to differentiate altered regions from normal regions is appropriate with CARAT, and provides a more accurate estimation of the true performance of CARAT. In this case, CARAT achieves perfect specificity of one and a very high specificity resulting in overall superior performance.

**Table 3:** Detailed information on the 69 SNPs with q-PCR result on SK-BR-3.

ID	Chr	Pos	2^ΔCt + 1^	CNAG	ID	Chr	Pos	2^ΔCt + 1^	CNAG
1715815	1	18690258	3.488	1	1730991	8	12219548	26.909	1
1677772	1	18961201	5.667	1	1736241	8	12247290	25.15	1
1701237	1	18961224	7.26	1	1733286	8	12247313	16.336	1
1754488	1	19467510	3.824	0	1705485	8	12284860	14.52	1
1682316	2	10879223	2.144	1	1642293	8	12466876	15.428	1
1721952	2	11234432	2.54	0	1642509	8	12706003	1.588	1
1685376	3	2910083	2.056	1	1710395	8	12781129	12.376	1
1739241	3	54136934	1.599	1	1756158	8	12793955	14.566	1
1740943	3	17277166	1.464	1	1655411	8	13167929	0.05	1
1724728	4	9565339	1.155	1	1745005	8	13167938	1.079	1
1684152	4	9939264	0.878	1	1688950	8	13935299	1.092	0
1691466	4	18055558	0.961	1	1707862	9	28313673	1.735	1
1750183	4	36828550	1.055	1	1753803	10	93029981	1.253	1
1741115	4	10257358	4.408	0	1662598	10	95045955	0.953	0
1752629	4	10359355	3.122	1	1703209	12	38583007	3.249	0
1688092	4	15453342	2.467	1	1736669	12	41456018	2.01	0
1678384	4	17883743	5.948	1	1704347	13	10715383	3.358	1
1744079	5	15244637	5.483	1	1745652	15	78297093	2.27	1
1720887	7	11014665	3.084	1	1671362	17	10226391	1.27	0
1702115	7	12400720	5.116	1	1682916	17	37814459	29.293	1
1718607	7	12669364	4.571	1	1693987	17	39301708	14.672	1
1757769	8	17939677	1.542	1	1754640	17	40022109	4.865	1
1665304	8	71285928	19.126	1	1757956	17	42235557	1.037	0
1688836	8	75872813	3.289	1	1718017	17	48767363	1.091	1
1751239	8	78708089	4.098	1	1644936	18	5903606	2.003	1
1663022	8	79625135	9.142	0	1731333	18	47360984	0.7	1
1685422	8	87105878	6.207	1	1707435	18	48017003	0.433	1
1684480	8	87106029	6.229	1	1664936	18	48130851	0.476	1
1726250	8	89504951	10.056	1	1666836	18	48130957	0.498	1
1644190	8	90597245	5.169	1	1727821	18	48131045	0.622	1
1666582	8	10542849	0.572	1	1670177	19	20287003	0.227	1
1649454	8	10566581	0.428	0	1679882	19	20533429	0.086	1
1700191	8	11201815	32.706	1	1653046	19	23871401	0.025	0
1746553	8	12034217	13.881	0	1739377	20	10000010	7.198	0
1691086	8	12055400	10.465	1					

Note:

^1^2^ΔCt + 1^: theoretical estimate of total copy number using QPCR

^2^CNAG: "1" means included in the CNAG analysis (total 55); "0" means excluded from CNAG analysis (total 14) because the SNP resides on a restriction fragment of less than 500 bp.

**Table 4:** Comparison among CARAT, dCHIP and CNAG using QPCR results.

Method	Stage	Sensitivity	Specificity
CARAT	Single Point	0.956 (0.932)	0.444 (0.4)
	100 kb Smoothing	0.957 (0.898)	0.556 (0.6)
	Tree Partitioning	0.957 (0.932)	0.778 (0.8)
	Tree Partitioning (p-value)	0.957 (0.881)	1 (1)
dCHIP	Single Point	0.783 (0.780)	0.333 (0.4)
	10 Points Median	0.864 (0.913)	0.7 (0.667)
	HMM	0.978 (0.898)	0.778 (0.8)
CNAG	Single Point	0.609	1
	10 points Mean	0.522	1
	HMM	0.717	1

Note: SNPs that have no copy number alterations relative to the diploid state (true negative SNPs) are defined by a QPCR derived copy number between 1.5 and 2.5. SNPs that have copy number alterations (true positive SNPs) are defined by a QPCR derived copy number less than 1.5 or greater than 2.5. There are in total 10 true negatives among all 69 SNPs. For the 55 SNPs that are common to CNAG, there are 9 true negatives. The calculations of sensitivity and specificity compare QPCR values to algorithm outputs in which algorithm-defined true negatives are the SNPs with an estimated copy number between 1.5 and 2.5 and algorithm-defined true positives are the SNPs with an estimated copy number less than 1.5 or greater than 2.5. In the CARAT tree-partitioning (p-value) comparison, the algorithm defined negative SNPs are those with p-value > 0.005 and algorithm defined positive SNPs are those with p-value < 0.005. Numerical values without parentheses are derived from comparisons using only the 55 SNPs that are in common with CNAG while the numbers inside parentheses are derived from comparisons using all 69 SNPs.

**Figure 5:**
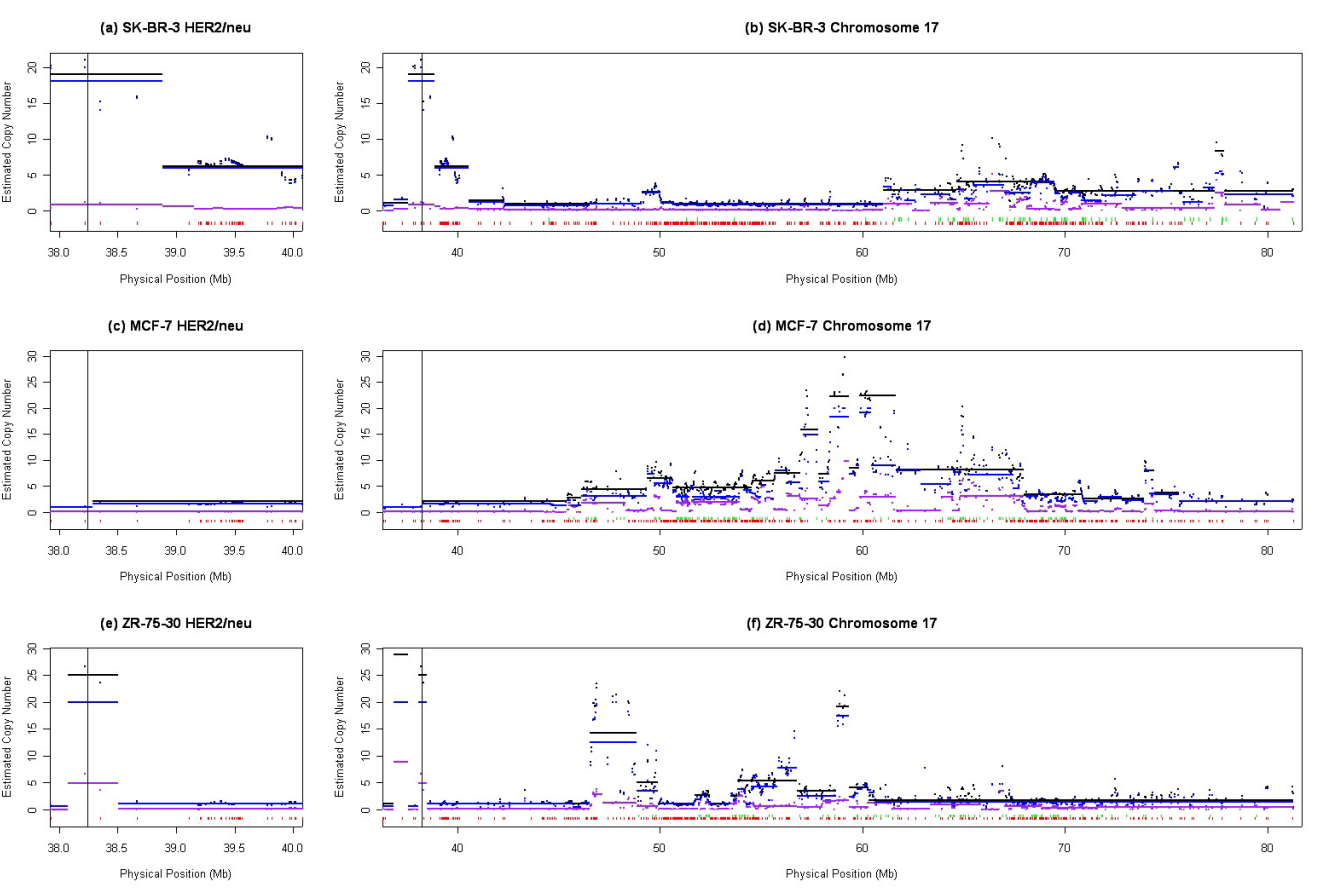
Three human breast cancer cell lines are represented by panels a-b (SK-BR-3), panels c-d (MCF-7), and panels e-f (ZR-75-30). The X-axis in all six panels is the physical position of SNPs along chromosome 17. The vertical lines just above the X-axis of each panel represent heterozygous (green) and homozygous (red) genotype calls. The Y-axis in all six panels is the estimated copy number. The points are derived from the kernel smoothing step and the solid horizontal lines are derived from the regression tree. Black colored lines indicate total copy number, the blue colored lines indicate the allele with the higher copy number estimate and the purple colored lines indicate the allele with the lower copy estimate. The vertical black line proximal to 40 Mb indicates the location of the HER2/neu gene. The panels on the left (panels a, c, and e) show an enlarged view of the genomic region harboring HER2/neu while the panels on the right (panels b, d, and f) show a larger view of the chromosome.

Additional verification of DNA copy number changes detected by CARAT was done using the highly characterized region on chromosome 17q12 harboring the ERBB2 (HER2/neu) proto-oncogene that is amplified in nearly 30% of breast cancers [48]. Figure 5 shows a comparison of chromosome 17 for three human breast cancer cell lines. The genomic region near HER2/neu appears amplified in the two cancer cell lines SK-BR-3 (panel a) and ZR-75-30 (panel e) with moderate to very strong significance (significance data not shown) and does not appear amplified in MCF-7 (panel c). This is consistent with published CGH results that show SK-BR-3 and ZR-75-30, but not MCF-7, contain gains in 17q12 [49] as well as with ERBB2-specific FISH showing amplification in SK-BR-3 (45 signals per cell) but not MCF-7 (2.5 signals per cell) [50]. Quantitative PCR was carried out with a HER2/neu primer pair and confirmed the copy number increase in two of the three cell lines (Table 5). The estimated HER2/neu copy number by QPCR for SK-BR-3, MCF-7, and ZR-75-30 is 12.4, 0.8, and 27.7 respectively. While the array set does not contain SNPs within the HER2/neu gene, the SNPs which flank the locus are SNPs 1720794 and 1738376. CARAT results for these SNPs are also summarized in Table 5 and confirm that the region surrounding HER2/NEU is amplified in two of the three cell lines. In Figure 5 all three cell lines show LOH in this region. Based on CARAT, MCF-7 shows one copy loss at the HER2/neu locus itself and proximal to the locus while there is no apparent copy number change distal to the locus. Additionally, SK-BR-3 and ZR-75-30 both show differential amplification of one allele relative to the other, resulting in allelic imbalance. These regions also serve to underscore how genotypic information can complement copy number information in the detection of complex structural alterations in regions exhibiting LOH. In Figure 5 panels b, d and f, results from CARAT are also consistent with additional regional copy-number increases observed by CGH using metaphase chromosomes in MCF-7 (17q22-q24; ~47.5–68.4 Mb), SK-BR-3 (17q24-qter; ~59.9––78.8 Mb), and ZR-75-30 (17cen-q24; ~22.8–68.4 Mb) [49].

**Table 5:** Comparison of QPCR and CARAT on HER2/neu locus across three cell lines.

HER2/NEU Region on Chr 17					
Sample	SNP 1720794 12.48 kb proximal		HER2/neu 38231.31–38259.79 kb	SNP 1738376 91.64 kb distal	
	CARAT	p-val	2^ΔCt + 1^	CARAT	p-val

SK-BR-3	19.0	<10^-20^	12.4	19.0	<10^-20^
ZR-75-30	25.1	<10^-20^	27.7	25.1	<10^-20^
MCF-7	1.0	0.0003	0.8	2.1	0.567

Note:

^1^2^ΔCt + 1^: theoretical estimate of total copy number using QPCR

^2^CARAT: copy number estimated by CARAT

^3^p-val: The statistical significance of the copy number alteration. It is derived from the algorithm by comparing the target sample to a reference set consisting of normal individuals

We chose 9 SNPs distributed across five different chromosomes for TaqMan analysis as an independent verification of allelic copy number information. These SNPs were identified by CARAT and represent various types of alterations. Allelic copy number results from CARAT and TaqMan for these SNPs across two cell lines are summarized in Table 6. TaqMan reactions for each SNP were done with genomic DNA from SK-BR-3 and ZR-75-30 as well as with normal DNA samples representing AA, AB, and BB genotypes that serve as positive controls for allele dosage. There are a total of 36 allele-specific copy number estimates when combining results for nine SNPs from the two cancer cell lines on both alleles. In general, there is a high linear correlation between the allelic copy number estimates using the algorithm and the allelic copy number derived from TaqMan reactions (Cor = 0.87). Among the 36 data points, there are 12 examples with a TaqMan-determined copy number lower than 0.5 and thus may indicate the loss of an allele. 10 out of these 12 examples also show a CARAT copy number estimation lower than 0.5, indicating a strong consistency between the two methods. These 12 examples can be further separated into four categories: (1) normal homozygous SNP (one allele present in two copies, the other allele absent), which includes SNP 1724728 and SNP 1736669 from ZR-75-30; (2) homozygous deletion (both alleles absent), which includes SNP 1670177 from SK-BR-3; (2) hemizygous deletion (one allele absent, one allele present at one copy), which includes SNPs 1724728 and 1718017 from SK-BR-3 and SNPs 1726250, 112706 and 1670177 from ZR-75-30; (4) hemizgyous deletion and one allele amplification (one allele absent and the other amplified), which includes SNP 1700191 from both samples, and SNP 1693987 from SK-BR-3. There are also 9 examples with a TaqMan-determined copy number higher than 2.5 indicating putative allelic amplification; all of these 9 examples also have a CARAT copy number estimation higher than 2.5. Some examples are explained by category (4) described above, while the remaining examples all appear as asymmetric amplifications (one allele remains intact, one allele amplified), including SNPs 1726250, 1746553, 1710029 from SK-BR-3 and SNPs 1710029 and 1718017 from ZR-75-30. When the TaqMan-determined total copy number is less than 1 or greater than 3, the CARAT determined p-value is always very significant (< 0.0001) with a single exception of SNP 112706 from ZR-75-30 (p-value 0.002).

**Table 6:** Allele specific Taqman results

		Sample	SK-BR-3				ZR-75-30			
SNPID	Chr	Pos	P-Value	CN_A	CN_B	DM Call	P-Value	CN_A	CN_B	DM Call

1724728	4	9565339	0.0001	0.89 (0.85)	0.22 (0.0)	AA	0.0006	1.77 (1.5)	0.19 (0.02)	NC
1726250	8	89504951	<10^-20^	0.83 (0.97)	14.83 (10.8)	BB	0.141	0.43 (0.0)	0.84 (1.21)	BB
1700191	8	112018158	5.03 × 10^-11^	0.34 (0.08)	4.57 (5.54)	BB	3.77 × 10^-19^	0.81 (0.01)	8.99 (8.93)	BB
1746553	8	120342175	1.72 × 10^-20^	18.45 (14.32)	1.17 (1.43)	AA	8.09 × 10^-20^	10.44 (5.7)	1.19 (0.68)	AA
1710029	8	121176983	1.53 × 10^-20^	19.05 (35.5)	3.30 (1.02)	AA	3.61 × 10^-20^	13.60 (9.1)	2.46 (0.77)	NC
1736669	12	41456018	0.843	1.10 (1.08)	0.90 (0.99)	NC	0.075	1.68 (1.61)	1.39 (0.03)	AA
1693987	17	39301708	1.38 × 10^-16^	6.20 (10.64)	0.28 (0.28)	AA	0.002	0.08 (0.0)	1.06 (0.66)	NC
1718017	17	48767363	0.0001	0.93 (0.9)	0.15 (0.0)	NC	5.96 × 10^-19^	6.53 (5.58)	2.04 (1.12)	NC
1670177	19	20287003	1.01 × 10^-9^	0.0 (0.0)	0.03 (0.0)	NC	0.310	0.11 (0.36)	1.47 (0.74)	NC

Note: WGSA results for 9 SNPs are shown in columns labeled CN_A (copy number of A allele) and CN_B (copy number of B allele). Allelic copy number estimates by TaqMan reactions are shown in parentheses. DM Call refers to the genotype call made using the Dynamic Model algorithm (DM) using a confidence rank score of 0.05. The VIC reporter dye was measuring the A allele for all SNPs except SNP 1710029 (B allele). The FAM reporter dye was measuring the B allele for all SNPs except SNP 1710029 (A allele).

**Figure 6:**
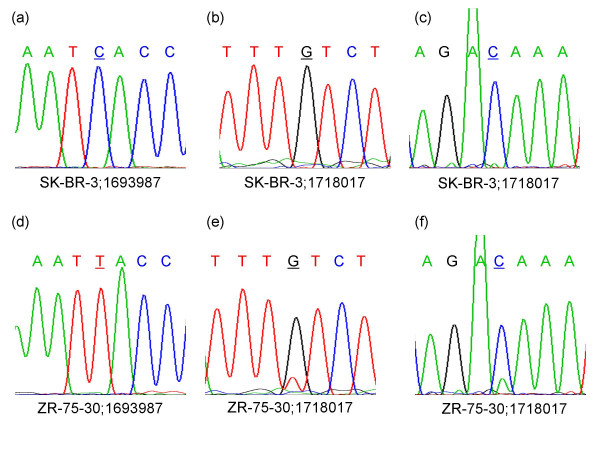
DNA sequencing traces surrounding the polymorphic nucleotide are shown in each panel. The SNP corresponds to the underlined base. Panel a and d represent tracings using the forward sequencing primer for SNP 1693987. Panels b and e represent tracings using the forward sequencing primer for SNP 1718017 while panels c and f represent tracings using the reverse sequencing primer for SNP 1718017.

In addition to allelic TaqMan reactions, direct DNA sequencing was carried out on PCR amplicons from both cell lines for seven of the SNPs. Several examples are shown in Figure 6. Panels a and d represent sequence traces using a forward primer for SNP 1693987 from SK-BR-3 and ZR-75-30 respectively. The polymorphic nucleotide in the sense strand is either C (allele A) or T (allele B). SK-BR-3 shows a clear blue peak representing the A allele while ZR-75-30 shows a clear red peak representing the B allele. Both of these base calls are consistent with the predominant allele identified by both CARAT and TaqMan. The copy number of allele B (SNP 1693987) from SK-BR-3 is below 0.5 copies based on CARAT and TaqMan while the copy number of allele A is greater than six with both methods. The DNA sequence trace however does not detect the presence of the minor allele. In contrast, the signal from the minor allele can be detected in the case of SNP 1718017 as shown in panels b, c, e, and f. The polymorphic nucleotide in the sense strand is either G (allele A) or T (allele B). Sequence traces using the forward primer show that in both cell lines the major allele is the A allele (G) as indicated by the black peak. However, ZR-75-30 also shows a smaller red peak indicating the presence of allele B (T). The tracings using the reverse primer also confirm the major allele is the A allele (G) in both cell lines, and ZR-75-30 again shows a minor green peak corresponding to allele B (T). There is no clear detection of the minor allele in the sequence traces from SK-BR-3 (panel b and c) which is consistent with both the CARAT (0.15 copies) and TaqMan (0 copies) results. In ZR-75-30, the ratio of the A allele peak height to the B allele peak height is 3.3 in the forward traces and 4.9 in the reverse traces, which are in general agreement with the allele ratios of 3.2 by CARAT and 5.0 by TaqMan. Thus the DNA sequencing results for this SNP confirm the CARAT and TaqMan results which suggested that allele B was present in at least one to two copies in ZR-75-30 but not in SK-BR-3.

## Conclusion

We have developed an algorithm CARAT used in conjunction with the GeneChip^® ^Mapping 100 K Set that provides accurate copy number estimates in an allele-specific manner. This algorithm makes use of the highly accurate genotypic information across a set of normal individuals to identify probes with strong allele-specific dose responses. The copy number estimation is accompanied by a significance score derived by a comparison to a reference set of normal individuals. Kernel smoothing with a Gaussian kernel and a relatively small bandwidth of 100kb is applied on the individual estimates in an attempt to achieve a balance between resolution and noise reduction. Regression trees are applied at the final stage as a method to partition the genome into regions that share the same copy number and to assign an overall copy number and significance to every region that alters from the diploid state. This partitioning step further reduces the random variability from SNP to SNP and increases the interpretability of the output. Although regression trees are conceptually simple, they solve the complex issue of how to define genomic regions with similar alterations. The assumption under regression trees is that different regions of the feature space have a constant outcome. With a series of recursive binary splits, they efficiently and accurately stratify the feature space into groups such that the random deviation from the fitted constant is minimized [51]. In the application of regression trees to DNA copy number analysis, the feature space is one dimensional and corresponds to the physical location on the chromosome while the outcome is the unknown copy number. The non-parametric nature of the tree method thus uncouples it from the many assumptions associated with particular distributions, which is especially appropriate for this array-platform since the behavior of probe intensity can be complex and difficult to summarize. The kernel smoothing step used for noise reduction and the tree partitioning step used for genome segmentation provide an optimal combination that renders high performance along with simple interpretation of the output [52]. This combination of information allows genomic alterations that lead to allelic imbalance to be characterized in a manner that is not currently possible by approaches such as CGH. Additionally, allelic copy number potentially allows examples of both whole chromosome and segmental uniparental disomy to be identified as well as genome-wide assessments of monoallelic amplification [53].

There are a number of alternative statistical methods that have been used to analyze array data for the purpose of copy number variation detection. Several approaches have used Hidden Markov Models (HMMs) [1,47,54,55]. Although in general the Markov chain framework does fit genome-wide copy number variation, determination of the specific parameters in the model can depend on the patterns of variation in the samples. Thus the performance hinges on how well the actual distribution of copy number variation from experimental samples such as cancer cells, which is largely unknown, agrees with the distribution hypothesized by the model. In this study we compared CARAT with two methods that use HMMs, namely dCHIP and CNAG. The performance between dCHIP and CARAT is similar, while CNAG tends to bias towards the normal diploid stage. In addition, dCHIP can not offer allele specific information in contrast to CARAT and CNAG. However, the allele specific estimation in CNAG is only feasible in matched pairs of samples and then only considers those SNPs that are called heterozygous in the normal matched sample. CARAT is free of these constraints and allele specific copy number can be estimated on any SNP with any sample.

Additional approaches include change-point analysis [56,57] or posterior log likelihood [58] to partition the genome into normal versus changed regions. These approaches assume that the intensity variability of probes corresponding to sub-regions of the genome is similar. However, using WGSA and the high density arrays, we observe substantial variation in the intensities of different SNPs. This can result from differences in SNP probe sequences as well as the restriction fragment target sequences. Regression on the probe GC content and the restriction fragment length stabilizes SNP variability and improves sample-to-sample comparability. In addition, the use of a large normal reference set enables the intensity distribution on diploid genomes to be directly estimated at an individual SNP level, thereby improving the accuracy of the model. There is also an algorithm that uses a hierarchical clustering scheme along the chromosome to identify changes. Here the signal threshold is set by directly controlling the false discovery rate (FDR), providing researchers with a high level of confidence regarding their findings [59]. The challenge with such an approach is that a desirable FDR level can preclude the detection of moderate changes that only span a short stretch of the genome. This issue is also relevant to our algorithm in that the p-value threshold which separates significance from insignificance is determined empirically with the test set of normal individuals and with ROC analyses using the 1X to 5X samples; however, there still exists a balance between detection power versus false positive rate. In addition, kernel smoothing across neighboring SNPs can sacrifice single point resolution. The smoothing window chosen is 100kb with a Gaussian kernel where the points near the window boundary has minimum weight, rendering an average resolution of no lower than 100kb. Although this resolution is high compared to traditional CGH, it is nevertheless sub-optimal compared to the average of 30kb resolution of single point analysis. These issues in part should be off-set by new advancements that allow the resolution of the high density arrays to be further increased through a decrease in feature size and increase in target DNA complexity resulting in the capability to simultaneously genotype over 500, 000 SNPs using a pair of arrays.

## Methods

### Cell lines & DNA samples

All human breast cancer cell lines (MCF-7, SK-BR-3, and ZR-75-30) were obtained from American Type Culture Collection (ATCC). Genomic DNAs were isolated using QIAGEN QIAmp DNA Blood Mini Kit. DNA samples used as controls in allelic TaqMan analysis as well as DNA samples derived from cell lines containing 3X (NA04626), 4X (NA01416), and 5X (NA06061) chromosomes were purchased from NIGMS Human Genetic Cell Repository, Coriell Institute for Medical Research (Camden, NJ).

### WGSA

The whole genome sampling assay (WGSA) was performed using an earlier version of the final protocol. Briefly, 250 ng genomic DNA is digested in 20 μl with 10 U of either Xba I or Hind III restriction enzyme (New England Biolabs) at 37°C for 2 hr followed by heat inactivation at 70°C for 20 min. The digested DNA is ligated in 25 μl with 0.25 μM Xba I adaptors (5'-ATTATGAGCACGACAGACGCCTGATCT-3' and 5'-pCTAGAGATCAGGCGTCTGTCGTGCTCATAA-3') or Hind III adaptors (5'-ATTATGAGCACGACAGACGCCTGATCA-3' and 5'-pAGCTAGATCAGGCGTCTGTCGTGCTCATAA-3') and 250 units T4 DNA ligase (New England Biolabs) at 16°C for 2 hr followed by heat inactivation at 70°C for 20 min. DNA amplification is carried out by PCR under the following conditions: each 100 μl reaction contains 25 ng adapter-ligated genomic DNA, 1 μM primer (5'-ATTATGAGCACGACAGACGCCTGATCT-3'), 300 μM dNTPs, 1 mM MgSO_4_, 5 U *Pfx *polymerase (Invitrogen Corporation) in 1× *Pfx *Amplification buffer with 1× PCR enhancer (Invitrogen Corporation). Thermal cycling is performed with 94°C for 3 min, followed by 30 cycles of 94°C/30 sec, 60°C/45 sec, 68°C/1 min, and a final extension at 68°C for 7 min. PCR products are purified and concentrated with a QIAGEN mini-elute plate and then spectrophotometrically quantitated using absorbance at 260 nm. 40 μg PCR products are fragmented in 55 μl with 0.2 units DNase I (Affymetrix) at 37°C for 30 min, followed by heat inactivation at 95°C for 15 min. The fragmented DNA products are labeled in 70 μl reactions containing 1× TdT buffer with 105 units TdT (Promega) and 0.214 mM DLR (Affymetrix) at 37°C for 2 hr, followed by heat inactivation at 95°C for 15 min. DNA hybridization to the GeneChip^® ^Human Mapping 50 K Array Xba 240 and GeneChip^® ^Human Mapping 50 K Array Hind 240, washing, staining, and scanning were performed exactly as the manufacturers' instructions (Affymetrix). SNP genotype calls are made automatically using a likelihood-based model [60]

### Quantitative PCR and TaqMan assays

Quantitative PCR was performed using ABI Prism 7700 Sequence Detection System (ABI). PCR primers were designed by using Primer Express 1.5 software (ABI) and were synthesized by Operon Biotechnologies, Inc. Reactions were prepared using the SYBR-Green PCR Core Reagents kit (ABI). 69 autosomal SNPs were selected and tested. 25 μl reactions containing 25 ng genomic DNA were set up for each SNP. Normal human genomic DNA was purchased from Roche Applied Sciences. Conditions for amplification were as follows: 1 cycle of 50°C for 2 min, followed by 1 cycle of 95°C for 10 min, then followed by 35 cycles of 95°C for 20 sec, 56°C for 30 sec, and 72°C for 30 sec. Threshold cycle numbers were obtained by using Sequence Detector v1.7a software. For all 69 SNPs, Roche human genomic DNA was used as the normal control. All reactions were done in duplicate and threshold cycle numbers were averaged. DNA amounts were measured by UV spectrophotometry and were normalized to LINE-1 elements [19]. Relative quantitation was carried out using the comparative Ct method (ABI User Bulletin #2, 1997). Quantitative PCR assays for HER2/NEU were done as described except that the annealing temperature was 60°C. The primer sequences used for HER2/neu were (Fw) 5'-GAACTGGTGTATGCAGATTGC-3' and (Rv) 5'-AGCAAGAGTCCCCATCCTA-3'.

Primers and probes for allelic TaqMan analysis of 9 SNPs were ordered via the Assays-by-Design Service (ABI). TaqMan reactions contained 10 ng genomic DNA in a 25 μl reaction volume containing 1.25 U of Taq Gold DNA polymerase, 5 μM MgCl_2_, 250 μM of dNTPs, 1 μM each of PCR primer, and both the FAM and VIC labeled TaqMan probes for the SNP at 0.1 μM final concentration. The amplification conditions consisted of an initial incubation step at 95°C for 10 min, followed by 40 cycles at 92°C for 15 sec, 60°C for 1 min using an ABI Prism 7700 sequence detection system. The DNA amounts were normalized to LINE-1 elements. Each SNP was tested with three normal DNA samples that represented AA, AB, and BB genotypes. We estimated the allele-specific copy number with a linear model:

copy number = *η*_0 _+ *η*_1 _× 2^ΔCt ^    (1)

The parameters of such a model (i.e. *η*_*0 *_and *η*_*1*_) were estimated using the three normal samples that represented AA, AB and BB genotypes; "copy number" is the inherent two, one, or zero doses of the A allele and zero, one, or two doses of the B allele of the AA, AB, and BB genotypes from the corresponding normal samples; "ΔCt" is the TaqMan Ct difference between samples. There are in total 18 such models being fit (9 SNPs × 2 alleles per SNP). In general this linear framework fits very well with a mean R-square value of 0.993 and standard deviation of 0.009 across all 18 models. After *η*_*0 *_and *η*_*1 *_have been estimated for each allele of the nine SNPs, these 18 models are used to predict copy number from the Ct values associated with the experimental samples (SK-BR-3 and ZR-75-30).

### DNA sequence analysis

PCR primer pairs were designed for a subset of the SNPs. The primer pair sequences (Fw 5'-3'/Rv 5'-3') were SNP1724728 (GCTGAGGCTCTGGGAGTTC/ATGGAACTGCTGGAGGTTTG); SNP1726250 (ACATGGGCTGCAATATCCTC/GGGAGGTGGAAGAGAAAACC); SNP1700191 (GGGCAAAGGATCTGAATAAGC/CACATGCAGGTTTTTGTGTG); SNP1710029 (GACTGCCACAGTGGAAAGG/CCGTAGGCCTTCACTAGCAG); SNP1693987 (TTTTGGCCTTTGAGGCTATG/GGGTTCACCTTCCACACTTG); SNP1718017 (GAATCAGGTCACCAACATGG/AGTTCACAGCAAAGCACCAG); SNP1670177 (CCTCACAAAGAAGATTTGACCTG/TTGTCTTTCGGTCTTTGTGG). PCR products were sequenced by dideoxy DNA sequencing using the individual PCR primers as sequencing primers. Sequencing chromatograms were visualized using Chromas 2.3.

### CNAG and dCHIP

The following samples were used as a reference set during CNAG [61] analysis: NA17011, NA17101, NA17115, NA17201, and NA17214. The following samples were used as a reference set during dCHIP [62] analysis: NA15029, NA15385, NA15590, NA17011, NA17052, NA17053, NA17101, NA17115, NA17144, NA17172, NA17201, NA17214, NA17253, and NA17279. Default parameter settings are used for both methods.

### Data analysis

#### Intensity transformation and standardization

**Figure 7:**
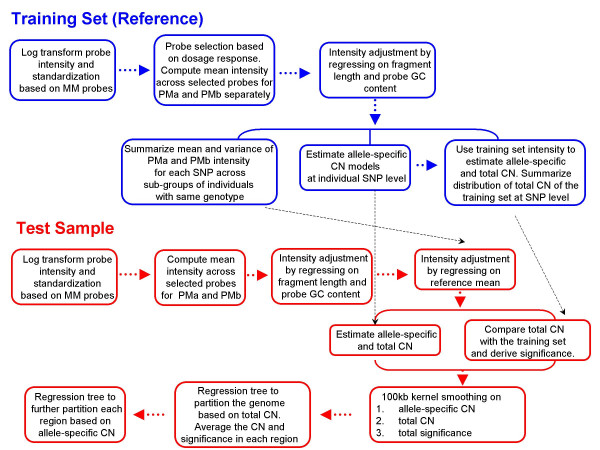
The CARAT algorithm is summarized as a flow chart, indicating the major steps in both the training set and the test set. "CN" refers to copy number. The black dotted line indicates how and where the information from the training set is used in the test set.

The basic premise underlying copy number estimation is that the natural log-transformed chip intensity, following adjustments on SNP-specific affinity and non-specific hybridization, is linearly related to the natural log of the DNA target copy number:

ln(*C *+ *δ*_*m*_) = *α*_*m*,0 _+ *α*_*m*,1 _(*I*_*m *_- *A*_*m*_) + *ε *    (2)

where *m *= *1*,....,*M *is the SNP index, *I*_*m *_is the natural log-transformed probe intensity on SNP *m*, *α*_*m*,0_, which is always a negative value, represents the quantity to be subtracted due to the SNP-specific optical background, *α*_*m,1 *_is the scaling factor, *δ*_m _is the non-specific hybridization, *C *is the DNA target concentration (i.e. copy number), *A*_*m *_is the affinity term determined by probe and target fragment sequences, and *ε *is the random noise. The allele specific copy number estimation (Eq 9 and 10) is based on this fundamental assumption. The only major difference is the affinity term *A*_*m*_in Eq 2, which has already been estimated and regressed out using a quadratic regression model with probe GC content and fragment length as the predictors (Eq 5 and 6). To better understand the details of the method, the main steps of the algorithm are summarized in a flowchart (Figure 7). The algorithm implements two rounds of standardization (Eq 3, 7, and 8). The first is applied on the natural log-transformed raw intensity (Eq 3) and establishes the comparability across samples. The second is applied prior to the copy number estimation, and realigns the target intensity according to the mean from the reference pool (Eq 7 and 8), thereby eliminating any systematic intensity shift that has not been adjusted by the previous standardization (Eq 3) or the affinity-based correction (Eq 5 and 6). This algorithm also employs a probe selection procedure (Eq 4) following the first round of standardization in which only probes that show a strong dosage response across samples (as described in Eq 2) are selected for further analysis. Kernel smoothing is applied on the estimated copy number at the level of individual SNPs to further reduce the experimental and technical noise. Regression trees are applied on the smoothed result to partition each chromosome into regions with different copy numbers, to assign significance to each region, and to increase the interpretability of the overall results. All the parameters in CARAT are optimized using a training set with 128 individuals. The training set (Coriell Repositories) consists of 42 African Americans, 20 Asians, 42 Caucasians and 24 samples from the polymorphism discovery panel [63]. Among them, 71 are females and 57 are males. The information from the training set including the intensity and genotype are publicly available upon request. Researchers can also use their own training set for CARAT.

Each SNP on the 100 K array set is represented by 40 unique features (probes): 10 perfect match (PM) probes and 10 mismatch (MM) probes for both the A and B alleles. The natural log-transformation of the raw intensity is first applied at the probe level for all SNPs. After the transformation, standardization is performed based on MM probe intensities that best represent background signals. This achieves a standard Gaussian distribution of the background intensity to increase the comparability across chips. For each array with a single DNA sample, background intensity is defined as the MMa probe intensity for all SNPs with 'BB' genotype calls and the MMb probe intensity for all SNPs with 'AA' genotype calls. All PMa probes are linearly transformed such that under the same transformation the MMa probes for SNPs with 'BB' genotype calls have a variance of one and a mean of zero; all PMb probes are linearly transformed such that under the same transformation the MMb probe intensity on SNPs with 'AA' genotype calls have a variance of one and a mean of zero.

Sa,lmn=ln⁡(PMa,lmn)−μ^a,lσ^a,l,Sb,lmn=ln⁡(PMb,lmn)−μ^b,lσ^b,l     (3)
 MathType@MTEF@5@5@+=feaafiart1ev1aaatCvAUfKttLearuWrP9MDH5MBPbIqV92AaeXatLxBI9gBaebbnrfifHhDYfgasaacH8akY=wiFfYdH8Gipec8Eeeu0xXdbba9frFj0=OqFfea0dXdd9vqai=hGuQ8kuc9pgc9s8qqaq=dirpe0xb9q8qiLsFr0=vr0=vr0dc8meaabaqaciaacaGaaeqabaqabeGadaaakeaafaqabeqacaaabaGaem4uam1aaSbaaSqaaiabdggaHjabcYcaSiabdYgaSjabd2gaTjabd6gaUbqabaGccqGH9aqpdaWcaaqaaiGbcYgaSjabc6gaUjabcIcaOiabdcfaqjabd2eannaaBaaaleaacqWGHbqycqGGSaalcqWGSbaBcqWGTbqBcqWGUbGBaeqaaOGaeiykaKIaeyOeI0ccciGaf8hVd0MbaKaadaWgaaWcbaGaemyyaeMaeiilaWIaemiBaWgabeaaaOqaaiqb=n8aZzaajaWaaSbaaSqaaiabdggaHjabcYcaSiabdYgaSbqabaaaaOGaeiilaWcabaGaem4uam1aaSbaaSqaaiabdkgaIjabcYcaSiabdYgaSjabd2gaTjabd6gaUbqabaGccqGH9aqpdaWcaaqaaiGbcYgaSjabc6gaUjabcIcaOiabdcfaqjabd2eannaaBaaaleaacqWGIbGycqGGSaalcqWGSbaBcqWGTbqBcqWGUbGBaeqaaOGaeiykaKIaeyOeI0Iaf8hVd0MbaKaadaWgaaWcbaGaemOyaiMaeiilaWIaemiBaWgabeaaaOqaaiqb=n8aZzaajaWaaSbaaSqaaiabdkgaIjabcYcaSiabdYgaSbqabaaaaaaakiaaxMaacaWLjaWaaeWaaeaacqaIZaWmaiaawIcacaGLPaaaaaa@75A8@

*with *μ^a,l
 MathType@MTEF@5@5@+=feaafiart1ev1aaatCvAUfKttLearuWrP9MDH5MBPbIqV92AaeXatLxBI9gBaebbnrfifHhDYfgasaacH8akY=wiFfYdH8Gipec8Eeeu0xXdbba9frFj0=OqFfea0dXdd9vqai=hGuQ8kuc9pgc9s8qqaq=dirpe0xb9q8qiLsFr0=vr0=vr0dc8meaabaqaciaacaGaaeqabaqabeGadaaakeaaiiGacuWF8oqBgaqcamaaBaaaleaacqWGHbqycqGGSaalcqWGSbaBaeqaaaaa@3231@*and *σ^a,l
 MathType@MTEF@5@5@+=feaafiart1ev1aaatCvAUfKttLearuWrP9MDH5MBPbIqV92AaeXatLxBI9gBaebbnrfifHhDYfgasaacH8akY=wiFfYdH8Gipec8Eeeu0xXdbba9frFj0=OqFfea0dXdd9vqai=hGuQ8kuc9pgc9s8qqaq=dirpe0xb9q8qiLsFr0=vr0=vr0dc8meaabaqaciaacaGaaeqabaqabeGadaaakeaaiiGacuWFdpWCgaqcamaaBaaaleaacqWGHbqycqGGSaalcqWGSbaBaeqaaaaa@323E@*the sample estimation under the assumption*

In (*MM*_*a, lmn *_| *SNPm is autosomal and called genotype BB on sample l*) ~ *N *(*μ*_*a,l*_, *σ*_*a,l*_)

*with *μ^b,l
 MathType@MTEF@5@5@+=feaafiart1ev1aaatCvAUfKttLearuWrP9MDH5MBPbIqV92AaeXatLxBI9gBaebbnrfifHhDYfgasaacH8akY=wiFfYdH8Gipec8Eeeu0xXdbba9frFj0=OqFfea0dXdd9vqai=hGuQ8kuc9pgc9s8qqaq=dirpe0xb9q8qiLsFr0=vr0=vr0dc8meaabaqaciaacaGaaeqabaqabeGadaaakeaaiiGacuWF8oqBgaqcamaaBaaaleaacqWGIbGycqGGSaalcqWGSbaBaeqaaaaa@3233@*and *σ^b,l
 MathType@MTEF@5@5@+=feaafiart1ev1aaatCvAUfKttLearuWrP9MDH5MBPbIqV92AaeXatLxBI9gBaebbnrfifHhDYfgasaacH8akY=wiFfYdH8Gipec8Eeeu0xXdbba9frFj0=OqFfea0dXdd9vqai=hGuQ8kuc9pgc9s8qqaq=dirpe0xb9q8qiLsFr0=vr0=vr0dc8meaabaqaciaacaGaaeqabaqabeGadaaakeaaiiGacuWFdpWCgaqcamaaBaaaleaacqWGIbGycqGGSaalcqWGSbaBaeqaaaaa@3240@*the sample estimation under the assumption*

In (*MM*_*b, lmn *_| *SNPm is autosomal and called genotype AA on sample l*) ~ *N *(*μ*_*b,l*_, *σ*_*b,l*_)

*l = 1,..., L *is the sample index; *m = 1,..., M *is the SNP index; *n = 1,..., N *is the probe index. μ^a,l
 MathType@MTEF@5@5@+=feaafiart1ev1aaatCvAUfKttLearuWrP9MDH5MBPbIqV92AaeXatLxBI9gBaebbnrfifHhDYfgasaacH8akY=wiFfYdH8Gipec8Eeeu0xXdbba9frFj0=OqFfea0dXdd9vqai=hGuQ8kuc9pgc9s8qqaq=dirpe0xb9q8qiLsFr0=vr0=vr0dc8meaabaqaciaacaGaaeqabaqabeGadaaakeaaiiGacuWF8oqBgaqcamaaBaaaleaacqWGHbqycqGGSaalcqWGSbaBaeqaaaaa@3231@, μ^b,l
 MathType@MTEF@5@5@+=feaafiart1ev1aaatCvAUfKttLearuWrP9MDH5MBPbIqV92AaeXatLxBI9gBaebbnrfifHhDYfgasaacH8akY=wiFfYdH8Gipec8Eeeu0xXdbba9frFj0=OqFfea0dXdd9vqai=hGuQ8kuc9pgc9s8qqaq=dirpe0xb9q8qiLsFr0=vr0=vr0dc8meaabaqaciaacaGaaeqabaqabeGadaaakeaaiiGacuWF8oqBgaqcamaaBaaaleaacqWGIbGycqGGSaalcqWGSbaBaeqaaaaa@3233@, σ^a,l
 MathType@MTEF@5@5@+=feaafiart1ev1aaatCvAUfKttLearuWrP9MDH5MBPbIqV92AaeXatLxBI9gBaebbnrfifHhDYfgasaacH8akY=wiFfYdH8Gipec8Eeeu0xXdbba9frFj0=OqFfea0dXdd9vqai=hGuQ8kuc9pgc9s8qqaq=dirpe0xb9q8qiLsFr0=vr0=vr0dc8meaabaqaciaacaGaaeqabaqabeGadaaakeaaiiGacuWFdpWCgaqcamaaBaaaleaacqWGHbqycqGGSaalcqWGSbaBaeqaaaaa@323E@, σ^b,l
 MathType@MTEF@5@5@+=feaafiart1ev1aaatCvAUfKttLearuWrP9MDH5MBPbIqV92AaeXatLxBI9gBaebbnrfifHhDYfgasaacH8akY=wiFfYdH8Gipec8Eeeu0xXdbba9frFj0=OqFfea0dXdd9vqai=hGuQ8kuc9pgc9s8qqaq=dirpe0xb9q8qiLsFr0=vr0=vr0dc8meaabaqaciaacaGaaeqabaqabeGadaaakeaaiiGacuWFdpWCgaqcamaaBaaaleaacqWGIbGycqGGSaalcqWGSbaBaeqaaaaa@3240@ are sample specific parameters; and are subject to change for any future experiments. Following natural log-transformation and standardization, the 20 PM probe intensities in conjunction with the genotype information is then analyzed for copy number information

#### Probe Selection

PM probes which display a strong dosage response are selected for use in the algorithm. Each SNP has three possible genotypes: AA, AB and BB, which each respectively contains two, one, or zero doses of the A allele and zero, one, or two doses of the B allele. This provides an inherent positive control to examine dosage performance at the individual probe level on a SNP-by-SNP basis. Probe intensity information from the normal reference set is compared with genotypic information from the same individuals. Features with a linear correlation greater than 0.6 between the known allelic dosages based on the genotype calls and the probe intensity are selected. Intensity across selected probes is averaged and used in subsequent calculations.

*A*_*m *_= *{ n *| *Cor *> *0.6 between S*_*a;rmn *_*and genotype G*_*rm *_*r *= *1,..., R is the reference set}*

*B*_*m *_= *{ n *| *Cor *> *0.6 between S*_*b;rmn *_*and genotype G*_*rm *_*r *= *1,..., R is the reference set}*

S¯a,lm=∑n∈AmSa,lmn#{Am};S¯b,lm=∑n∈BmSb,lmn#{Bm}     (4)
 MathType@MTEF@5@5@+=feaafiart1ev1aaatCvAUfKttLearuWrP9MDH5MBPbIqV92AaeXatLxBI9gBaebbnrfifHhDYfgasaacH8akY=wiFfYdH8Gipec8Eeeu0xXdbba9frFj0=OqFfea0dXdd9vqai=hGuQ8kuc9pgc9s8qqaq=dirpe0xb9q8qiLsFr0=vr0=vr0dc8meaabaqaciaacaGaaeqabaqabeGadaaakeaafaqabeqacaaabaGafm4uamLbaebadaWgaaWcbaGaemyyaeMaeiilaWIaemiBaWMaemyBa0gabeaakiabg2da9maalaaabaWaaabuaeaacqWGtbWudaWgaaWcbaGaemyyaeMaeiilaWIaemiBaWMaemyBa0MaemOBa4gabeaaaeaacqWGUbGBcqGHiiIZcqWGbbqqdaWgaaadbaGaemyBa0gabeaaaSqab0GaeyyeIuoaaOqaaiabcocaJiabcUha7jabdgeabnaaBaaaleaacqWGTbqBaeqaaOGaeiyFa0haaiabcUda7aqaaiqbdofatzaaraWaaSbaaSqaaiabdkgaIjabcYcaSiabdYgaSjabd2gaTbqabaGccqGH9aqpdaWcaaqaamaaqafabaGaem4uam1aaSbaaSqaaiabdkgaIjabcYcaSiabdYgaSjabd2gaTjabd6gaUbqabaaabaGaemOBa4MaeyicI4SaemOqai0aaSbaaWqaaiabd2gaTbqabaaaleqaniabggHiLdaakeaacqGGJaWicqGG7bWEcqWGcbGqdaWgaaWcbaGaemyBa0gabeaakiabc2ha9baaaaGaaCzcaiaaxMaadaqadaqaaiabisda0aGaayjkaiaawMcaaaaa@6C2A@

SNPs that do not have at least one selected probe for both PMa and PMb probe sets were excluded from further analysis. *A*_*m *_and *B*_*m *_are parameters determined by the training set and are fixed for any given training set.

#### Regression on probe GC content and restriction fragment length

Variation of the SNP intensities can in part be explained by properties of the probe and restriction fragment sequences [47,64]. These properties include but are not limited to the length and GC content of the restriction fragment target, GC content of the probe sequences, and secondary structure of the probe and target sequences. An evaluation of these factors identified the GC content of the probes and the restriction fragment length as main contributors to variability in probe intensities using the 100 K WGSA assay. Linear regression, which included linear and square terms of both variables, was applied to reduce the intensity variations.

S¯a,l=(S¯a,l1,S¯a,l2,…,S¯a,lm,…,S¯a,lM); S¯b,l=(S¯b,l1,S¯b,l2,…,S¯b,lm,…,S¯b,lM)S¯a,l=βa,0+βa,1+Xa,1+βa,2Xa,12+βa,3X2+βa,4X22+εaS¯b,l=βb,0+βb,1Xb,1+βb,2Xb,12+βb,3X2+βb,4X22+εb     (5)
 MathType@MTEF@5@5@+=feaafiart1ev1aaatCvAUfKttLearuWrP9MDH5MBPbIqV92AaeXatLxBI9gBaebbnrfifHhDYfgasaacH8akY=wiFfYdH8Gipec8Eeeu0xXdbba9frFj0=OqFfea0dXdd9vqai=hGuQ8kuc9pgc9s8qqaq=dirpe0xb9q8qiLsFr0=vr0=vr0dc8meaabaqaciaacaGaaeqabaqabeGadaaakeaafaqaaeWabaaabaGafm4uamLbaebadaWgaaWcbaGaemyyaeMaeiilaWIaemiBaWgabeaakiabg2da9iabcIcaOiqbdofatzaaraWaaSbaaSqaaiabdggaHjabcYcaSiabdYgaSjabigdaXaqabaGccqGGSaalcuWGtbWugaqeamaaBaaaleaacqWGHbqycqGGSaalcqWGSbaBcqaIYaGmaeqaaOGaeiilaWIaeSOjGSKaeiilaWIafm4uamLbaebadaWgaaWcbaGaemyyaeMaeiilaWIaemiBaWMaemyBa0gabeaakiabcYcaSiablAciljabcYcaSiqbdofatzaaraWaaSbaaSqaaiabdggaHjabcYcaSiabdYgaSjabd2eanbqabaGccqGGPaqkcqGG7aWocqqGGaaicuWGtbWugaqeamaaBaaaleaacqWGIbGycqGGSaalcqWGSbaBaeqaaOGaeyypa0JaeiikaGIafm4uamLbaebadaWgaaWcbaGaemOyaiMaeiilaWIaemiBaWMaeGymaedabeaakiabcYcaSiqbdofatzaaraWaaSbaaSqaaiabdkgaIjabcYcaSiabdYgaSjabikdaYaqabaGccqGGSaalcqWIMaYscqGGSaalcuWGtbWugaqeamaaBaaaleaacqWGIbGycqGGSaalcqWGSbaBcqWGTbqBaeqaaOGaeiilaWIaeSOjGSKaeiilaWIafm4uamLbaebadaWgaaWcbaGaemOyaiMaeiilaWIaemiBaWMaemyta0eabeaakiabcMcaPaqaaiqbdofatzaaraWaaSbaaSqaaiabdggaHjabcYcaSiabdYgaSbqabaGccqGH9aqpiiGacqWFYoGydaWgaaWcbaGaemyyaeMaeiilaWIaeGimaadabeaakiabgUcaRiab=j7aInaaBaaaleaacqWGHbqycqGGSaalcqaIXaqmaeqaaOGaey4kaSIaemiwaG1aaSbaaSqaaiabdggaHjabcYcaSiabigdaXaqabaGccqGHRaWkcqWFYoGydaWgaaWcbaGaemyyaeMaeiilaWIaeGOmaidabeaakiabdIfaynaaDaaaleaacqWGHbqycqGGSaalcqaIXaqmaeaacqaIYaGmaaGccqGHRaWkcqWFYoGydaWgaaWcbaGaemyyaeMaeiilaWIaeG4mamdabeaakiabdIfaynaaBaaaleaacqaIYaGmaeqaaOGaey4kaSIae8NSdi2aaSbaaSqaaiabdggaHjabcYcaSiabisda0aqabaGccqWGybawdaqhaaWcbaGaeGOmaidabaGaeGOmaidaaOGaey4kaSIae8xTdu2aaSbaaSqaaiabdggaHbqabaaakeaacuWGtbWugaqeamaaBaaaleaacqWGIbGycqGGSaalcqWGSbaBaeqaaOGaeyypa0Jae8NSdi2aaSbaaSqaaiabdkgaIjabcYcaSiabicdaWaqabaGccqGHRaWkcqWFYoGydaWgaaWcbaGaemOyaiMaeiilaWIaeGymaedabeaakiabdIfaynaaBaaaleaacqWGIbGycqGGSaalcqaIXaqmaeqaaOGaey4kaSIae8NSdi2aaSbaaSqaaiabdkgaIjabcYcaSiabikdaYaqabaGccqWGybawdaqhaaWcbaGaemOyaiMaeiilaWIaeGymaedabaGaeGOmaidaaOGaey4kaSIae8NSdi2aaSbaaSqaaiabdkgaIjabcYcaSiabiodaZaqabaGccqWGybawdaWgaaWcbaGaeGOmaidabeaakiabgUcaRiab=j7aInaaBaaaleaacqWGIbGycqGGSaalcqaI0aanaeqaaOGaemiwaG1aa0baaSqaaiabikdaYaqaaiabikdaYaaakiabgUcaRiab=v7aLnaaBaaaleaacqWGIbGyaeqaaaaakiaaxMaacaWLjaWaaeWaaeaacqaI1aqnaiaawIcacaGLPaaaaaa@ED36@

*X*_*a,1 *_is the probe GC content averaged across the selected PMa probes; *X*_*b,1 *_is the probe GC content averaged across the selected PMb probes; and *X*_*2 *_is the restriction fragment length. The regression coefficients are sample-specific and thus are re-estimated for each new sample.

S˜a,l=β^a,0+ε^aS˜b,l=β^b,0+ε^bS˜a,l=(S˜a,l1,S˜a,l2,…,S˜a,lm,…,S˜a,lM); S˜b,l=(S˜b,l1,S˜b,l2,…,S˜b,lm,…,S˜b,lM)     (6)
 MathType@MTEF@5@5@+=feaafiart1ev1aaatCvAUfKttLearuWrP9MDH5MBPbIqV92AaeXatLxBI9gBaebbnrfifHhDYfgasaacH8akY=wiFfYdH8Gipec8Eeeu0xXdbba9frFj0=OqFfea0dXdd9vqai=hGuQ8kuc9pgc9s8qqaq=dirpe0xb9q8qiLsFr0=vr0=vr0dc8meaabaqaciaacaGaaeqabaqabeGadaaakqaabeqaauaabeqabiaaaeaacuWGtbWugaacamaaBaaaleaacqWGHbqycqGGSaalcqWGSbaBaeqaaOGaeyypa0dcciGaf8NSdiMbaKaadaWgaaWcbaGaemyyaeMaeiilaWIaeGimaadabeaakiabgUcaRiqb=v7aLzaajaWaaSbaaSqaaiabdggaHbqabaaakeaacuWGtbWugaacamaaBaaaleaacqWGIbGycqGGSaalcqWGSbaBaeqaaOGaeyypa0Jaf8NSdiMbaKaadaWgaaWcbaGaemOyaiMaeiilaWIaeGimaadabeaakiabgUcaRiqb=v7aLzaajaWaaSbaaSqaaiabdkgaIbqabaaaaaGcbaGafm4uamLbaGaadaWgaaWcbaGaemyyaeMaeiilaWIaemiBaWgabeaakiabg2da9iabcIcaOiqbdofatzaaiaWaaSbaaSqaaiabdggaHjabcYcaSiabdYgaSjabigdaXaqabaGccqGGSaalcuWGtbWugaacamaaBaaaleaacqWGHbqycqGGSaalcqWGSbaBcqaIYaGmaeqaaOGaeiilaWIaeSOjGSKaeiilaWIafm4uamLbaGaadaWgaaWcbaGaemyyaeMaeiilaWIaemiBaWMaemyBa0gabeaakiabcYcaSiablAciljabcYcaSiqbdofatzaaiaWaaSbaaSqaaiabdggaHjabcYcaSiabdYgaSjabd2eanbqabaGccqGGPaqkcqGG7aWocqqGGaaicuWGtbWugaacamaaBaaaleaacqWGIbGycqGGSaalcqWGSbaBaeqaaOGaeyypa0JaeiikaGIafm4uamLbaGaadaWgaaWcbaGaemOyaiMaeiilaWIaemiBaWMaeGymaedabeaakiabcYcaSiqbdofatzaaiaWaaSbaaSqaaiabdkgaIjabcYcaSiabdYgaSjabikdaYaqabaGccqGGSaalcqWIMaYscqGGSaalcuWGtbWugaacamaaBaaaleaacqWGIbGycqGGSaalcqWGSbaBcqWGTbqBaeqaaOGaeiilaWIaeSOjGSKaeiilaWIafm4uamLbaGaadaWgaaWcbaGaemOyaiMaeiilaWIaemiBaWMaemyta0eabeaakiabcMcaPiaaxMaacaWLjaWaaeWaaeaacqaI2aGnaiaawIcacaGLPaaaaaaa@9E1C@

The residuals plus the constant term were used as adjusted intensity in the coming steps with the effects due to the probes and the fragment being regressed out.

#### Regression adjustment

Following the standardization and regression on the probe GC content and the restriction fragment length, a further correction of systematic intensity deviations was done by a regression on the reference set mean intensities. The reference mean intensity for a given probe set (PMa or PMb) and genotype (AA, AB or BB) was calculated for each SNP. For a given test sample, two regressions are performed in this adjustment step: one for PMa and one for PMb. In each regression, the PM intensity on the test sample across all SNPs is regressed against the average PM intensity of the reference samples that shares the same genotype as the test sample. With the estimated regression coefficients, the test sample is linearly transformed by subtracting the intercept then dividing by the slope such that after the transformation, the regression line of the test sample intensity against the average reference intensity is *Y *= *X*.

*R*_*m*,*AA *_= *{r *| *G*_*rm *_= *AA; r *= *1, ..., R};     R*_*m*,*AB *_= *{r | G*_*rm *_= *AB; r *= *1*, ..., *R};*

*R*_*m*,*BB *_= *{r *| *G*_*rm *_= *BB; r *= *1, ..., R}; R*_*m*,*all *_= *{r | G*_*rm *_= *AA, AB or BB ; r *= *1*, ..., *R};*

S˜a,l=αa,0+αa,1×Ua,l+ε;S˜b,l=αb,0+αb,1×Ub,l+εUa,l=(Ua,l1,Ua,l2,…,Ua,lm,…,Ua,lM); Ub,l=(Ub,l1,Ub,l2,…,Ub,lm,…,Ub,lM)     (7)
 MathType@MTEF@5@5@+=feaafiart1ev1aaatCvAUfKttLearuWrP9MDH5MBPbIqV92AaeXatLxBI9gBaebbnrfifHhDYfgasaacH8akY=wiFfYdH8Gipec8Eeeu0xXdbba9frFj0=OqFfea0dXdd9vqai=hGuQ8kuc9pgc9s8qqaq=dirpe0xb9q8qiLsFr0=vr0=vr0dc8meaabaqaciaacaGaaeqabaqabeGadaaakqaabeqaauaabeqabiaaaeaacuWGtbWugaacamaaBaaaleaacqWGHbqycqGGSaalcqWGSbaBaeqaaOGaeyypa0dcciGae8xSde2aaSbaaSqaaiabdggaHjabcYcaSiabicdaWaqabaGccqGHRaWkcqWFXoqydaWgaaWcbaGaemyyaeMaeiilaWIaeGymaedabeaakiabgEna0kabdwfavnaaBaaaleaacqWGHbqycqGGSaalcqWGSbaBaeqaaOGaey4kaSIae8xTduMaei4oaSdabaGafm4uamLbaGaadaWgaaWcbaGaemOyaiMaeiilaWIaemiBaWgabeaakiabg2da9iab=f7aHnaaBaaaleaacqWGIbGycqGGSaalcqaIWaamaeqaaOGaey4kaSIae8xSde2aaSbaaSqaaiabdkgaIjabcYcaSiabigdaXaqabaGccqGHxdaTcqWGvbqvdaWgaaWcbaGaemOyaiMaeiilaWIaemiBaWgabeaakiabgUcaRiab=v7aLbaaaeaacqWGvbqvdaWgaaWcbaGaemyyaeMaeiilaWIaemiBaWgabeaakiabg2da9iabcIcaOiabdwfavnaaBaaaleaacqWGHbqycqGGSaalcqWGSbaBcqaIXaqmaeqaaOGaeiilaWIaemyvau1aaSbaaSqaaiabdggaHjabcYcaSiabdYgaSjabikdaYaqabaGccqGGSaalcqWIMaYscqGGSaalcqWGvbqvdaWgaaWcbaGaemyyaeMaeiilaWIaemiBaWMaemyBa0gabeaakiabcYcaSiablAciljabcYcaSiabdwfavnaaBaaaleaacqWGHbqycqGGSaalcqWGSbaBcqWGnbqtaeqaaOGaeiykaKIaei4oaSJaeeiiaaIaemyvau1aaSbaaSqaaiabdkgaIjabcYcaSiabdYgaSbqabaGccqGH9aqpcqGGOaakcqWGvbqvdaWgaaWcbaGaemOyaiMaeiilaWIaemiBaWMaeGymaedabeaakiabcYcaSiabdwfavnaaBaaaleaacqWGIbGycqGGSaalcqWGSbaBcqaIYaGmaeqaaOGaeiilaWIaeSOjGSKaeiilaWIaemyvau1aaSbaaSqaaiabdkgaIjabcYcaSiabdYgaSjabd2gaTbqabaGccqGGSaalcqWIMaYscqGGSaalcqWGvbqvdaWgaaWcbaGaemOyaiMaeiilaWIaemiBaWMaemyta0eabeaakiabcMcaPiaaxMaacaWLjaWaaeWaaeaacqaI3aWnaiaawIcacaGLPaaaaaaa@B51C@

Ua,lm={∑r∈Rm,AAS˜a,rm#{Rm,AA}Glm=AA∑r∈Rm,ABS˜a,rm#{Rm,AB}Glm=AB∑r∈Rm,BBS˜a,rm#{Rm,BB}Glm=BB∑r∈Rm,allS˜a,rm#{Rm,all}Glm=No callUb,lm={∑r∈Rm,AAS˜b,rm#{Rm,AA}Glm=AA∑r∈Rm,ABS˜b,rm#{Rm,AB}Glm=AB∑r∈Rm,BBS˜b,rm#{Rm,BB}Glm=BB∑r∈Rm,allS˜b,rm#{Rm,all}Glm=No callIa,l=S˜a,l−α^a,0α^a,1;Ib,l=S˜b,l−α^b,0α^b,1     (8)Ia,l=(Ia,l1,Ia,l2,…,Ia,lm,…,Ia,lM); Ib,l=(Ib,l1,Ib,l2,…,Ib,lm,…,Ib,lM)
 MathType@MTEF@5@5@+=feaafiart1ev1aaatCvAUfKttLearuWrP9MDH5MBPbIqV92AaeXatLxBI9gBaebbnrfifHhDYfgasaacH8akY=wiFfYdH8Gipec8Eeeu0xXdbba9frFj0=OqFfea0dXdd9vqai=hGuQ8kuc9pgc9s8qqaq=dirpe0xb9q8qiLsFr0=vr0=vr0dc8meaabaqaciaacaGaaeqabaqabeGadaaakqGabeqaaa=gbaqbaeqabiGaaaqaaiabdwfavnaaBaaaleaacqWGHbqycqGGSaalcqWGSbaBcqWGTbqBaeqaaOGaeyypa0ZaaiqaaeaafaqabeabcaaaaeaadaWcaaqaamaaqafabaGafm4uamLbaGaadaWgaaWcbaGaemyyaeMaeiilaWIaemOCaiNaemyBa0gabeaaaeaacqWGYbGCcqGHiiIZcqWGsbGudaWgaaadbaGaemyBa0MaeiilaWIaemyqaeKaemyqaeeabeaaaSqab0GaeyyeIuoaaOqaaiabcocaJiabcUha7jabdkfasnaaBaaaleaacqWGTbqBcqGGSaalcqWGbbqqcqWGbbqqaeqaaOGaeiyFa0haaaqaaiabdEeahnaaBaaaleaacqWGSbaBcqWGTbqBaeqaaOGaeyypa0JaemyqaeKaemyqaeeabaWaaSaaaeaadaaeqbqaaiqbdofatzaaiaWaaSbaaSqaaiabdggaHjabcYcaSiabdkhaYjabd2gaTbqabaaabaGaemOCaiNaeyicI4SaemOuai1aaSbaaWqaaiabd2gaTjabcYcaSiabdgeabjabdkeacbqabaaaleqaniabggHiLdaakeaacqGGJaWicqGG7bWEcqWGsbGudaWgaaWcbaGaemyBa0MaeiilaWIaemyqaeKaemOqaieabeaakiabc2ha9baaaeaacqWGhbWrdaWgaaWcbaGaemiBaWMaemyBa0gabeaakiabg2da9iabdgeabjabdkeacbqaamaalaaabaWaaabuaeaacuWGtbWugaacamaaBaaaleaacqWGHbqycqGGSaalcqWGYbGCcqWGTbqBaeqaaaqaaiabdkhaYjabgIGiolabdkfasnaaBaaameaacqWGTbqBcqGGSaalcqWGcbGqcqWGcbGqaeqaaaWcbeqdcqGHris5aaGcbaGaei4iamIaei4EaSNaemOuai1aaSbaaSqaaiabd2gaTjabcYcaSiabdkeacjabdkeacbqabaGccqGG9bqFaaaabaGaem4raC0aaSbaaSqaaiabdYgaSjabd2gaTbqabaGccqGH9aqpcqWGcbGqcqWGcbGqaeaadaWcaaqaamaaqafabaGafm4uamLbaGaadaWgaaWcbaGaemyyaeMaeiilaWIaemOCaiNaemyBa0gabeaaaeaacqWGYbGCcqGHiiIZcqWGsbGudaWgaaadbaGaemyBa0MaeiilaWIaemyyaeMaemiBaWMaemiBaWgabeaaaSqab0GaeyyeIuoaaOqaaiabcocaJiabcUha7jabdkfasnaaBaaaleaacqWGTbqBcqGGSaalcqWGHbqycqWGSbaBcqWGSbaBaeqaaOGaeiyFa0haaaqaaiabdEeahnaaBaaaleaacqWGSbaBcqWGTbqBaeqaaOGaeyypa0JaemOta4Kaem4Ba8MaeeiiaaIaem4yamMaemyyaeMaemiBaWMaemiBaWgaaaGaay5EaaaabaGaemyvau1aaSbaaSqaaiabdkgaIjabcYcaSiabdYgaSjabd2gaTbqabaGccqGH9aqpdaGabaqaauaabeqaeiaaaaqaamaalaaabaWaaabuaeaacuWGtbWugaacamaaBaaaleaacqWGIbGycqGGSaalcqWGYbGCcqWGTbqBaeqaaaqaaiabdkhaYjabgIGiolabdkfasnaaBaaameaacqWGTbqBcqGGSaalcqWGbbqqcqWGbbqqaeqaaaWcbeqdcqGHris5aaGcbaGaei4iamIaei4EaSNaemOuai1aaSbaaSqaaiabd2gaTjabcYcaSiabdgeabjabdgeabbqabaGccqGG9bqFaaaabaGaem4raC0aaSbaaSqaaiabdYgaSjabd2gaTbqabaGccqGH9aqpcqWGbbqqcqWGbbqqaeaadaWcaaqaamaaqafabaGafm4uamLbaGaadaWgaaWcbaGaemOyaiMaeiilaWIaemOCaiNaemyBa0gabeaaaeaacqWGYbGCcqGHiiIZcqWGsbGudaWgaaadbaGaemyBa0MaeiilaWIaemyqaeKaemOqaieabeaaaSqab0GaeyyeIuoaaOqaaiabcocaJiabcUha7jabdkfasnaaBaaaleaacqWGTbqBcqGGSaalcqWGbbqqcqWGcbGqaeqaaOGaeiyFa0haaaqaaiabdEeahnaaBaaaleaacqWGSbaBcqWGTbqBaeqaaOGaeyypa0JaemyqaeKaemOqaieabaWaaSaaaeaadaaeqbqaaiqbdofatzaaiaWaaSbaaSqaaiabdkgaIjabcYcaSiabdkhaYjabd2gaTbqabaaabaGaemOCaiNaeyicI4SaemOuai1aaSbaaWqaaiabd2gaTjabcYcaSiabdkeacjabdkeacbqabaaaleqaniabggHiLdaakeaacqGGJaWicqGG7bWEcqWGsbGudaWgaaWcbaGaemyBa0MaeiilaWIaemOqaiKaemOqaieabeaakiabc2ha9baaaeaacqWGhbWrdaWgaaWcbaGaemiBaWMaemyBa0gabeaakiabg2da9iabdkeacjabdkeacbqaamaalaaabaWaaabuaeaacuWGtbWugaacamaaBaaaleaacqWGIbGycqGGSaalcqWGYbGCcqWGTbqBaeqaaaqaaiabdkhaYjabgIGiolabdkfasnaaBaaameaacqWGTbqBcqGGSaalcqWGHbqycqWGSbaBcqWGSbaBaeqaaaWcbeqdcqGHris5aaGcbaGaei4iamIaei4EaSNaemOuai1aaSbaaSqaaiabd2gaTjabcYcaSiabdggaHjabdYgaSjabdYgaSbqabaGccqGG9bqFaaaabaGaem4raC0aaSbaaSqaaiabdYgaSjabd2gaTbqabaGccqGH9aqpcqWGobGtcqWGVbWBcqqGGaaicqWGJbWycqWGHbqycqWGSbaBcqWGSbaBaaaacaGL7baaaeaacqWGjbqsdaWgaaWcbaGaemyyaeMaeiilaWIaemiBaWgabeaakiabg2da9maalaaabaGafm4uamLbaGaadaWgaaWcbaGaemyyaeMaeiilaWIaemiBaWgabeaakiabgkHiTGGaciqb=f7aHzaajaWaaSbaaSqaaiabdggaHjabcYcaSiabicdaWaqabaaakeaacuWFXoqygaqcamaaBaaaleaacqWGHbqycqGGSaalcqaIXaqmaeqaaaaakiabcUda7aqaaiabdMeajnaaBaaaleaacqWGIbGycqGGSaalcqWGSbaBaeqaaOGaeyypa0ZaaSaaaeaacuWGtbWugaacamaaBaaaleaacqWGIbGycqGGSaalcqWGSbaBaeqaaOGaeyOeI0Iaf8xSdeMbaKaadaWgaaWcbaGaemOyaiMaeiilaWIaeGimaadabeaaaOqaaiqb=f7aHzaajaWaaSbaaSqaaiabdkgaIjabcYcaSiabigdaXaqabaaaaaaakiaaxMaacaWLjaWaaeWaaeaacqaI4aaoaiaawIcacaGLPaaaaeaacqWGjbqsdaWgaaWcbaGaemyyaeMaeiilaWIaemiBaWgabeaakiabg2da9iabcIcaOiabdMeajnaaBaaaleaacqWGHbqycqGGSaalcqWGSbaBcqaIXaqmaeqaaOGaeiilaWIaemysaK0aaSbaaSqaaiabdggaHjabcYcaSiabdYgaSjabikdaYaqabaGccqGGSaalcqWIMaYscqGGSaalcqWGjbqsdaWgaaWcbaGaemyyaeMaeiilaWIaemiBaWMaemyBa0gabeaakiabcYcaSiablAciljabcYcaSiabdMeajnaaBaaaleaacqWGHbqycqGGSaalcqWGSbaBcqWGnbqtaeqaaOGaeiykaKIaei4oaSJaeeiiaaIaemysaK0aaSbaaSqaaiabdkgaIjabcYcaSiabdYgaSbqabaGccqGH9aqpcqGGOaakcqWGjbqsdaWgaaWcbaGaemOyaiMaeiilaWIaemiBaWMaeGymaedabeaakiabcYcaSiabdMeajnaaBaaaleaacqWGIbGycqGGSaalcqWGSbaBcqaIYaGmaeqaaOGaeiilaWIaeSOjGSKaeiilaWIaemysaK0aaSbaaSqaaiabdkgaIjabcYcaSiabdYgaSjabd2gaTbqabaGccqGGSaalcqWIMaYscqGGSaalcqWGjbqsdaWgaaWcbaGaemOyaiMaeiilaWIaemiBaWMaemyta0eabeaakiabcMcaPaaaaa@E28F@

Where *R*_*m*,*AA*_, *R*_*m*,*AB*_, *R*_*m*,*BB*_, *R*_*m*,*all *_are the corresponding subsets of the reference samples whose genotypes are "AA", "AB", "BB", and the union of the three groups on SNP *m, (m = 1 to M)*; *U*_*a,l *_and *U*_*b,l *_are two vectors of the average PMa, PMb intensity across all SNPs on reference samples that share the same genotype as the target sample *l; G*_*lm *_is the genotype of test sample *l *on SNP *m*; S˜a,rm
 MathType@MTEF@5@5@+=feaafiart1ev1aaatCvAUfKttLearuWrP9MDH5MBPbIqV92AaeXatLxBI9gBaebbnrfifHhDYfgasaacH8akY=wiFfYdH8Gipec8Eeeu0xXdbba9frFj0=OqFfea0dXdd9vqai=hGuQ8kuc9pgc9s8qqaq=dirpe0xb9q8qiLsFr0=vr0=vr0dc8meaabaqaciaacaGaaeqabaqabeGadaaakeaacuWGtbWugaacamaaBaaaleaacqWGHbqycqGGSaalcqWGYbGCcqWGTbqBaeqaaaaa@3311@ and S˜b,rm
 MathType@MTEF@5@5@+=feaafiart1ev1aaatCvAUfKttLearuWrP9MDH5MBPbIqV92AaeXatLxBI9gBaebbnrfifHhDYfgasaacH8akY=wiFfYdH8Gipec8Eeeu0xXdbba9frFj0=OqFfea0dXdd9vqai=hGuQ8kuc9pgc9s8qqaq=dirpe0xb9q8qiLsFr0=vr0=vr0dc8meaabaqaciaacaGaaeqabaqabeGadaaakeaacuWGtbWugaacamaaBaaaleaacqWGIbGycqGGSaalcqWGYbGCcqWGTbqBaeqaaaaa@3313@ are the PMa, PMb intensity of reference sample *r (r = 1 to R)*, SNP *m (m = 1 to M); *S˜a,l
 MathType@MTEF@5@5@+=feaafiart1ev1aaatCvAUfKttLearuWrP9MDH5MBPbIqV92AaeXatLxBI9gBaebbnrfifHhDYfgasaacH8akY=wiFfYdH8Gipec8Eeeu0xXdbba9frFj0=OqFfea0dXdd9vqai=hGuQ8kuc9pgc9s8qqaq=dirpe0xb9q8qiLsFr0=vr0=vr0dc8meaabaqaciaacaGaaeqabaqabeGadaaakeaacuWGtbWugaacamaaBaaaleaacqWGHbqycqGGSaalcqWGSbaBaeqaaaaa@31A2@ and S˜b,l
 MathType@MTEF@5@5@+=feaafiart1ev1aaatCvAUfKttLearuWrP9MDH5MBPbIqV92AaeXatLxBI9gBaebbnrfifHhDYfgasaacH8akY=wiFfYdH8Gipec8Eeeu0xXdbba9frFj0=OqFfea0dXdd9vqai=hGuQ8kuc9pgc9s8qqaq=dirpe0xb9q8qiLsFr0=vr0=vr0dc8meaabaqaciaacaGaaeqabaqabeGadaaakeaacuWGtbWugaacamaaBaaaleaacqWGIbGycqGGSaalcqWGSbaBaeqaaaaa@31A4@ are the PMa, PMb intensity of test sample *l *before this adjustment step; and *I*_*a*,*l *_and *I*_*b*,*l *_are the PMa, PMb intensity of test sample *l *after this adjustment step. The regression coefficients are sample dependent and thus are estimated for each specific sample.

#### Single point copy number prediction and significance calculation

A *ln*-*ln *model was used to estimate the copy number of each allele under the assumption that the natural log of the DNA target copy number has a linear relationship with the natural log-transformed intensity, where *r = 1,...,R *equals the reference set with an assumed diploid genome.

Ia,m=(Ia,1m,Ia,2m,…,Ia,rm,…,Ia,Rm); Ib,m=(Ib,1m,Ib,2m,…,Ib,rm,…,Ib,Rm)Ca,m=(Ca,1m,Ca,2m,…,Ca,rm,…,Ca,Rm); Cb,m=(Cb,1m,Cb,2m,…,Cb,rm,…,Cb,Rm)ln⁡(Ca,m+δa,m)=αa0,m+αa1,mIa,m+εa,mln⁡(Cb,m+δb,m)=αb0,m+αb1,mIb,m+εb,m     (9)
 MathType@MTEF@5@5@+=feaafiart1ev1aaatCvAUfKttLearuWrP9MDH5MBPbIqV92AaeXatLxBI9gBaebbnrfifHhDYfgasaacH8akY=wiFfYdH8Gipec8Eeeu0xXdbba9frFj0=OqFfea0dXdd9vqai=hGuQ8kuc9pgc9s8qqaq=dirpe0xb9q8qiLsFr0=vr0=vr0dc8meaabaqaciaacaGaaeqabaqabeGadaaakeaafaqaaeabbaaaaeaacqWGjbqsdaWgaaWcbaGaemyyaeMaeiilaWIaemyBa0gabeaakiabg2da9iabcIcaOiabdMeajnaaBaaaleaacqWGHbqycqGGSaalcqaIXaqmcqWGTbqBaeqaaOGaeiilaWIaemysaK0aaSbaaSqaaiabdggaHjabcYcaSiabikdaYiabd2gaTbqabaGccqGGSaalcqWIMaYscqGGSaalcqWGjbqsdaWgaaWcbaGaemyyaeMaeiilaWIaemOCaiNaemyBa0gabeaakiabcYcaSiablAciljabcYcaSiabdMeajnaaBaaaleaacqWGHbqycqGGSaalcqWGsbGucqWGTbqBaeqaaOGaeiykaKIaei4oaSJaeeiiaaIaemysaK0aaSbaaSqaaiabdkgaIjabcYcaSiabd2gaTbqabaGccqGH9aqpcqGGOaakcqWGjbqsdaWgaaWcbaGaemOyaiMaeiilaWIaeGymaeJaemyBa0gabeaakiabcYcaSiabdMeajnaaBaaaleaacqWGIbGycqGGSaalcqaIYaGmcqWGTbqBaeqaaOGaeiilaWIaeSOjGSKaeiilaWIaemysaK0aaSbaaSqaaiabdkgaIjabcYcaSiabdkhaYjabd2gaTbqabaGccqGGSaalcqWIMaYscqGGSaalcqWGjbqsdaWgaaWcbaGaemOyaiMaeiilaWIaemOuaiLaemyBa0gabeaakiabcMcaPaqaaiabdoeadnaaBaaaleaacqWGHbqycqGGSaalcqWGTbqBaeqaaOGaeyypa0JaeiikaGIaem4qam0aaSbaaSqaaiabdggaHjabcYcaSiabigdaXiabd2gaTbqabaGccqGGSaalcqWGdbWqdaWgaaWcbaGaemyyaeMaeiilaWIaeGOmaiJaemyBa0gabeaakiabcYcaSiablAciljabcYcaSiabdoeadnaaBaaaleaacqWGHbqycqGGSaalcqWGYbGCcqWGTbqBaeqaaOGaeiilaWIaeSOjGSKaeiilaWIaem4qam0aaSbaaSqaaiabdggaHjabcYcaSiabdkfasjabd2gaTbqabaGccqGGPaqkcqGG7aWocqqGGaaicqqGdbWqdaWgaaWcbaGaemOyaiMaeiilaWIaemyBa0gabeaakiabg2da9iabcIcaOiabdoeadnaaBaaaleaacqWGIbGycqGGSaalcqaIXaqmcqWGTbqBaeqaaOGaeiilaWIaem4qam0aaSbaaSqaaiabdkgaIjabcYcaSiabikdaYiabd2gaTbqabaGccqGGSaalcqWIMaYscqGGSaalcqWGdbWqdaWgaaWcbaGaemOyaiMaeiilaWIaemOCaiNaemyBa0gabeaakiabcYcaSiablAciljabcYcaSiabdoeadnaaBaaaleaacqWGIbGycqGGSaalcqWGsbGucqWGTbqBaeqaaOGaeiykaKcabaGagiiBaWMaeiOBa4MaeiikaGIaem4qam0aaSbaaSqaaiabdggaHjabcYcaSiabd2gaTbqabaGccqGHRaWkiiGacqWF0oazdaWgaaWcbaGaemyyaeMaeiilaWIaemyBa0gabeaakiabcMcaPiabg2da9iab=f7aHnaaBaaaleaacqWGHbqycqaIWaamcqGGSaalcqWGTbqBaeqaaOGaey4kaSIae8xSde2aaSbaaSqaaiabdggaHjabigdaXiabcYcaSiabd2gaTbqabaGccqWGjbqsdaWgaaWcbaGaemyyaeMaeiilaWIaemyBa0gabeaakiabgUcaRiab=v7aLnaaBaaaleaacqWGHbqycqGGSaalcqWGTbqBaeqaaaGcbaGagiiBaWMaeiOBa4MaeiikaGIaem4qam0aaSbaaSqaaiabdkgaIjabcYcaSiabd2gaTbqabaGccqGHRaWkcqWF0oazdaWgaaWcbaGaemOyaiMaeiilaWIaemyBa0gabeaakiabcMcaPiabg2da9iab=f7aHnaaBaaaleaacqWGIbGycqaIWaamcqGGSaalcqWGTbqBaeqaaOGaey4kaSIae8xSde2aaSbaaSqaaiabdkgaIjabigdaXiabcYcaSiabd2gaTbqabaGccqWGjbqsdaWgaaWcbaGaemOyaiMaeiilaWIaemyBa0gabeaakiabgUcaRiab=v7aLnaaBaaaleaacqWGIbGycqGGSaalcqWGTbqBaeqaaaaakiaaxMaacaWLjaWaaeWaaeaacqaI5aqoaiaawIcacaGLPaaaaaa@1F05@

The parameters in the formulas were estimated using the reference set and their known genotypes. *I*_*a,rm *_and *I*_*b,rm *_are the PMa, PMb intensity of reference sample *r *on SNP *m; C*_*a,rm *_and *C*_*b,rm *_are the known copy numbers on the A and B alleles of reference sample *r *on SNP *m*. Since the allelic copy number can be equal to zero, for each SNP *m*, two small positive numbers *δ*_*a,m *_and *δ*_*b,m *_which represent the non-specific hybridization that account for the baseline intensity, were added. The values of *δ*_*a,m *_and *δ*_*b,m *_were tested over a range of 0 to 5 with 0.01 increments and the value which generated the highest linear correlation between the natural log-transformed copy number *ln(C*_*a*,*m *_+ *δ*_*a*,*m*_*) *(*ln(C*_*b*,*m *_+ *δ*_*b*,*m*_*)*) and the natural log-transformed chip intensity *I*_*a*,*m *_(*I*_*b*,*m*_) were selected. SNPs with the highest correlation value < 0.8 are removed from further analysis. After *δ*_*a*,*m *_and *δ*_*b*,*m *_were fixed, The terms that represent the effect of optical background *α*_*a0*,*m*_, *α*_*b0*,*m*_, and the scaling factor *α*_*a1*,*m*_, *α*_*b1*,*m*_, were estimated using least square regression with the normal references as the training set. After the estimation, the final copy number equation is:

C^a,lm=max⁡(exp⁡(α^a1,mIa,lm+α^a0,m)−δ^a,m,0)C^b,lm=max⁡(exp⁡(α^b1,mIb,lm+α^b0,m)−δ^b,m,0)     (10)
 MathType@MTEF@5@5@+=feaafiart1ev1aaatCvAUfKttLearuWrP9MDH5MBPbIqV92AaeXatLxBI9gBaebbnrfifHhDYfgasaacH8akY=wiFfYdH8Gipec8Eeeu0xXdbba9frFj0=OqFfea0dXdd9vqai=hGuQ8kuc9pgc9s8qqaq=dirpe0xb9q8qiLsFr0=vr0=vr0dc8meaabaqaciaacaGaaeqabaqabeGadaaakeaafaqabeGabaaabaGafm4qamKbaKaadaWgaaWcbaGaemyyaeMaeiilaWIaemiBaWMaemyBa0gabeaakiabg2da9iGbc2gaTjabcggaHjabcIha4jabcIcaOiGbcwgaLjabcIha4jabcchaWjabcIcaOGGaciqb=f7aHzaajaWaaSbaaSqaaiabdggaHjabigdaXiabcYcaSiabd2gaTbqabaGccqWGjbqsdaWgaaWcbaGaemyyaeMaeiilaWIaemiBaWMaemyBa0gabeaakiabgUcaRiqb=f7aHzaajaWaaSbaaSqaaiabdggaHjabicdaWiabcYcaSiabd2gaTbqabaGccqGGPaqkcqGHsislcuWF0oazgaqcamaaBaaaleaacqWGHbqycqGGSaalcqWGTbqBaeqaaOGaeiilaWIaeGimaaJaeiykaKcabaGafm4qamKbaKaadaWgaaWcbaGaemOyaiMaeiilaWIaemiBaWMaemyBa0gabeaakiabg2da9iGbc2gaTjabcggaHjabcIha4jabcIcaOiGbcwgaLjabcIha4jabcchaWjabcIcaOiqb=f7aHzaajaWaaSbaaSqaaiabdkgaIjabigdaXiabcYcaSiabd2gaTbqabaGccqWGjbqsdaWgaaWcbaGaemOyaiMaeiilaWIaemiBaWMaemyBa0gabeaakiabgUcaRiqb=f7aHzaajaWaaSbaaSqaaiabdkgaIjabicdaWiabcYcaSiabd2gaTbqabaGccqGGPaqkcqGHsislcuWF0oazgaqcamaaBaaaleaacqWGIbGycqGGSaalcqWGTbqBaeqaaOGaeiilaWIaeGimaaJaeiykaKcaaiaaxMaacaWLjaWaaeWaaeaacqaIXaqmcqaIWaamaiaawIcacaGLPaaaaaa@8FA0@

All the parameters involved in the allele-specific copy number model are fixed with a given training set.

The copy number calculation is allele specific and SNP specific. The values for the two alleles can be summed to present the total copy number. The significance of the total copy number is calculated to identify putative amplifications and deletions. The reference samples are refitted into the *ln-ln *linear models and predicted total copy numbers are recorded. For a given SNP *m*, and a given reference sample *r (r = 1 to R)*, the predicted total copy number is:

C^t,rm=C^a,rm+C^b,rm=max⁡(exp⁡(α^a0,m+α^a1,mS˜a,rm)−δ^a,m,0)+max⁡(exp⁡(α^b0,m+α^b1,mS˜b,rm)−δ^b,m,0)     (11)
 MathType@MTEF@5@5@+=feaafiart1ev1aaatCvAUfKttLearuWrP9MDH5MBPbIqV92AaeXatLxBI9gBaebbnrfifHhDYfgasaacH8akY=wiFfYdH8Gipec8Eeeu0xXdbba9frFj0=OqFfea0dXdd9vqai=hGuQ8kuc9pgc9s8qqaq=dirpe0xb9q8qiLsFr0=vr0=vr0dc8meaabaqaciaacaGaaeqabaqabeGadaaakeaacuWGdbWqgaqcamaaBaaaleaacqWG0baDcqGGSaalcqWGYbGCcqWGTbqBaeqaaOGaeyypa0Jafm4qamKbaKaadaWgaaWcbaGaemyyaeMaeiilaWIaemOCaiNaemyBa0gabeaakiabgUcaRiqbdoeadzaajaWaaSbaaSqaaiabdkgaIjabcYcaSiabdkhaYjabd2gaTbqabaGccqGH9aqpcyGGTbqBcqGGHbqycqGG4baEcqGGOaakcyGGLbqzcqGG4baEcqGGWbaCcqGGOaakiiGacuWFXoqygaqcamaaBaaaleaacqWGHbqycqaIWaamcqGGSaalcqWGTbqBaeqaaOGaey4kaSIaf8xSdeMbaKaadaWgaaWcbaGaemyyaeMaeGymaeJaeiilaWIaemyBa0gabeaakiqbdofatzaaiaWaaSbaaSqaaiabdggaHjabcYcaSiabdkhaYjabd2gaTbqabaGccqGGPaqkcqGHsislcuWF0oazgaqcamaaBaaaleaacqWGHbqycqGGSaalcqWGTbqBaeqaaOGaeiilaWIaeGimaaJaeiykaKIaey4kaSIagiyBa0MaeiyyaeMaeiiEaGNaeiikaGIagiyzauMaeiiEaGNaeiiCaaNaeiikaGIaf8xSdeMbaKaadaWgaaWcbaGaemOyaiMaeGimaaJaeiilaWIaemyBa0gabeaakiabgUcaRiqb=f7aHzaajaWaaSbaaSqaaiabdkgaIjabigdaXiabcYcaSiabd2gaTbqabaGccuWGtbWugaacamaaBaaaleaacqWGIbGycqGGSaalcqWGYbGCcqWGTbqBaeqaaOGaeiykaKIaeyOeI0Iaf8hTdqMbaKaadaWgaaWcbaGaemOyaiMaeiilaWIaemyBa0gabeaakiabcYcaSiabicdaWiabcMcaPiaaxMaacaWLjaWaaeWaaeaacqaIXaqmcqaIXaqmaiaawIcacaGLPaaaaaa@9845@

For each SNP, there will be a range of variability across the normal reference samples based on their estimated copy numbers and such variability is summarized as the reference distribution under the Gaussian assumption. Target samples are compared to this reference distribution and significance is calculated accordingly [35].

C^t,m=(C^t,1m,C^t,2m,…,C^t,rm,…,C^t,Rm)C^t,m∼N(μtm,σtm2)μ^tm=1R∑r=1RC^t,rmσ^tm=1R−1∑r=1R(C^t,rm−μ^tm)2     (12)
 MathType@MTEF@5@5@+=feaafiart1ev1aaatCvAUfKttLearuWrP9MDH5MBPbIqV92AaeXatLxBI9gBaebbnrfifHhDYfgasaacH8akY=wiFfYdH8Gipec8Eeeu0xXdbba9frFj0=OqFfea0dXdd9vqai=hGuQ8kuc9pgc9s8qqaq=dirpe0xb9q8qiLsFr0=vr0=vr0dc8meaabaqaciaacaGaaeqabaqabeGadaaakeaafaqabeGabaaabaGafm4qamKbaKaadaWgaaWcbaGaemiDaqNaeiilaWIaemyBa0gabeaakiabg2da9iabcIcaOiqbdoeadzaajaWaaSbaaSqaaiabdsha0jabcYcaSiabigdaXiabd2gaTbqabaGccqGGSaalcuWGdbWqgaqcamaaBaaaleaacqWG0baDcqGGSaalcqaIYaGmcqWGTbqBaeqaaOGaeiilaWIaeSOjGSKaeiilaWIafm4qamKbaKaadaWgaaWcbaGaemiDaqNaeiilaWIaemOCaiNaemyBa0gabeaakiabcYcaSiablAciljabcYcaSiqbdoeadzaajaWaaSbaaSqaaiabdsha0jabcYcaSiabdkfasjabd2gaTbqabaGccqGGPaqkaeaafaqabeqadaaabaGafm4qamKbaKaadaWgaaWcbaGaemiDaqNaeiilaWIaemyBa0gabeaakiablYJi6iabd6eaojabcIcaOGGaciab=X7aTnaaBaaaleaacqWG0baDcqWGTbqBaeqaaOGaeiilaWIae83Wdm3aa0baaSqaaiabdsha0jabd2gaTbqaaiabikdaYaaakiabcMcaPaqaaiqb=X7aTzaajaWaaSbaaSqaaiabdsha0jabd2gaTbqabaGccqGH9aqpdaWcaaqaaiabigdaXaqaaiabdkfasbaadaaeWbqaaiqbdoeadzaajaWaaSbaaSqaaiabdsha0jabcYcaSiabdkhaYjabd2gaTbqabaaabaGaemOCaiNaeyypa0JaeGymaedabaGaemOuaifaniabggHiLdaakeaacuWFdpWCgaqcamaaBaaaleaacqWG0baDcqWGTbqBaeqaaOGaeyypa0ZaaOaaaeaadaWcaaqaaiabigdaXaqaaiabdkfasjabgkHiTiabigdaXaaadaaeWbqaaiabcIcaOiqbdoeadzaajaWaaSbaaSqaaiabdsha0jabcYcaSiabdkhaYjabd2gaTbqabaGccqGHsislcuWF8oqBgaqcamaaBaaaleaacqWG0baDcqWGTbqBaeqaaOGaeiykaKYaaWbaaSqabeaacqaIYaGmaaaabaGaemOCaiNaeyypa0JaeGymaedabaGaemOuaifaniabggHiLdaaleqaaaaaaaGccaWLjaGaaCzcamaabmaabaGaeGymaeJaeGOmaidacaGLOaGaayzkaaaaaa@A1F5@

For a given test sample *l *on SNP *m*, the total copy number estimate is:

C^t,lm=C^a,lm+C^b,lm=max⁡(exp⁡(α^a0,m+α^a1,mIa,lm)−δ^a,m,0)+max⁡(exp⁡(α^b0,m+α^b1,mIb,lm)−δ^b,m,0)     (13)
 MathType@MTEF@5@5@+=feaafiart1ev1aaatCvAUfKttLearuWrP9MDH5MBPbIqV92AaeXatLxBI9gBaebbnrfifHhDYfgasaacH8akY=wiFfYdH8Gipec8Eeeu0xXdbba9frFj0=OqFfea0dXdd9vqai=hGuQ8kuc9pgc9s8qqaq=dirpe0xb9q8qiLsFr0=vr0=vr0dc8meaabaqaciaacaGaaeqabaqabeGadaaakeaacuWGdbWqgaqcamaaBaaaleaacqWG0baDcqGGSaalcqWGSbaBcqWGTbqBaeqaaOGaeyypa0Jafm4qamKbaKaadaWgaaWcbaGaemyyaeMaeiilaWIaemiBaWMaemyBa0gabeaakiabgUcaRiqbdoeadzaajaWaaSbaaSqaaiabdkgaIjabcYcaSiabdYgaSjabd2gaTbqabaGccqGH9aqpcyGGTbqBcqGGHbqycqGG4baEcqGGOaakcyGGLbqzcqGG4baEcqGGWbaCcqGGOaakiiGacuWFXoqygaqcamaaBaaaleaacqWGHbqycqaIWaamcqGGSaalcqWGTbqBaeqaaOGaey4kaSIaf8xSdeMbaKaadaWgaaWcbaGaemyyaeMaeGymaeJaeiilaWIaemyBa0gabeaakiabdMeajnaaBaaaleaacqWGHbqycqGGSaalcqWGSbaBcqWGTbqBaeqaaOGaeiykaKIaeyOeI0Iaf8hTdqMbaKaadaWgaaWcbaGaemyyaeMaeiilaWIaemyBa0gabeaakiabcYcaSiabicdaWiabcMcaPiabgUcaRiGbc2gaTjabcggaHjabcIha4jabcIcaOiGbcwgaLjabcIha4jabcchaWjabcIcaOiqb=f7aHzaajaWaaSbaaSqaaiabdkgaIjabicdaWiabcYcaSiabd2gaTbqabaGccqGHRaWkcuWFXoqygaqcamaaBaaaleaacqWGIbGycqaIXaqmcqGGSaalcqWGTbqBaeqaaOGaemysaK0aaSbaaSqaaiabdkgaIjabcYcaSiabdYgaSjabd2gaTbqabaGccqGGPaqkcqGHsislcuWF0oazgaqcamaaBaaaleaacqWGIbGycqGGSaalcqWGTbqBaeqaaOGaeiilaWIaeGimaaJaeiykaKIaaCzcaiaaxMaadaqadaqaaiabigdaXiabiodaZaGaayjkaiaawMcaaaaa@97C7@

And the significance is calculated as:

plm=min⁡(1−Φ(C^t,lm−μ^tmσ^tm), Φ(C^t,lm−μ^tmσ^tm))     (14)
 MathType@MTEF@5@5@+=feaafiart1ev1aaatCvAUfKttLearuWrP9MDH5MBPbIqV92AaeXatLxBI9gBaebbnrfifHhDYfgasaacH8akY=wiFfYdH8Gipec8Eeeu0xXdbba9frFj0=OqFfea0dXdd9vqai=hGuQ8kuc9pgc9s8qqaq=dirpe0xb9q8qiLsFr0=vr0=vr0dc8meaabaqaciaacaGaaeqabaqabeGadaaakeaacqWGWbaCdaWgaaWcbaGaemiBaWMaemyBa0gabeaakiabg2da9iGbc2gaTjabcMgaPjabc6gaUjabcIcaOiabigdaXiabgkHiTiabfA6agjabcIcaOmaalaaabaGafm4qamKbaKaadaWgaaWcbaGaemiDaqNaeiilaWIaemiBaWMaemyBa0gabeaakiabgkHiTGGaciqb=X7aTzaajaWaaSbaaSqaaiabdsha0jabd2gaTbqabaaakeaacuWFdpWCgaqcamaaBaaaleaacqWG0baDcqWGTbqBaeqaaaaakiabcMcaPiabcYcaSiabbccaGiabfA6agjabcIcaOmaalaaabaGafm4qamKbaKaadaWgaaWcbaGaemiDaqNaeiilaWIaemiBaWMaemyBa0gabeaakiabgkHiTiqb=X7aTzaajaWaaSbaaSqaaiabdsha0jabd2gaTbqabaaakeaacuWFdpWCgaqcamaaBaaaleaacqWG0baDcqWGTbqBaeqaaaaakiabcMcaPiabcMcaPiaaxMaacaWLjaWaaeWaaeaacqaIXaqmcqaI0aanaiaawIcacaGLPaaaaaa@6898@

The significance at each SNP tests whether the copy number value associated with the SNP deviates from the diploid state.

#### Define regions with significant alterations

Before defining regions with significant alterations, kernel smoothing is applied to reduce the effect of outliers caused by inherent experimental error as well as the occasional true single-marker copy number variant. A bandwidth of 100Kb with a Gaussian kernel is applied on the total copy number, the significance associated with the total copy number (i.e. log10 transformed p-values), and the allele-specific copy number. The bandwidth is fixed for all the analyses. For allele specific copy number, smoothing is applied separately on the lower copy number estimate and the higher copy number estimate at each marker in an effort to present the phased data. In order to achieve a better estimation, putative regions of LOH are first identified, defined as more than k (k = 10) contiguous homozygous calls on the genome; intermittent "no calls" are allowed but not counted in k. In such regions, all markers, i.e. homozygous calls and no-calls, participate in the allele-specific smoothing. In all other regions, only markers with heterozygous genotypes are used for smoothing to prevent the underestimation of one strand and the overestimation of the other. In the idealized case of a perfect copy number prediction on a normal diploid region of the genome, there will be heterozygous SNP genotype calls interweaved with homozygous SNP genotype calls. For heterozygous calls the lower copy number estimation and the higher copy number estimation of the two alleles will be both close to one. For homozygous calls, the lower copy number estimation for one allele will be near zero and the higher copy number estimation for the other allele will be near two. If both homozygous calls and heterozygous calls are used for allele-specific copy number smoothing, then the single point estimation on the "lower-copy-number" strand will contain interweaved values close to either zero or one. After smoothing, the copy number will be lower than one. Similarly, for the alternate DNA strand, the single point estimation will contain values close to two or one and the smoothed values will therefore be higher than one. Thus using only heterozygous calls in these normal regions largely reduces such under (over) estimation. In regions with long stretches of homozygous calls, which rarely occurs randomly and is more likely caused by asymmetry between the two strands, it is more appropriate to use all the markers to do the allele-specific copy number smoothing.

After smoothing, regression trees [45] are applied with the physical location of each marker as the solo predictor and the natural-log transformed total copy number plus one as the outcome (adding one is done to avoid negative infinity in the case of a homozygous deletion). Log-transformation is used because heuristically the variation in intensity has been observed to increase with copy number; and log-transformation stabilizes the variance and better fits the regression tree framework. The complexity parameter is set to a small value cp = 0.0001 to ensure that a complex enough partition is tested and to ensure that splits which do not increase the overall R-squared value by 0.01% are not tested. In addition, regions with equal to or less than three points are not further split. After a complex enough partition is achieved, 10-fold cross-validation and the one-standard deviation rule are applied to prune the large tree back to an appropriate size to control for over-fitting. After the final partitioning, the average across a region is assigned as the copy number of that region (it will be a geometric average of the original copy number estimate since the regression tree is performed on the log-transformed copy number space); the significance of that region is the average of the log10 transformed p-values (deletion uses log10; amplification uses -log10). Within each chromosome, regions with overall non-significant p-values (p-value > 0.01) are merged with copy number information and the significance values are then updated under the assumption that they represent the same normal diploid state. For allele-specific copy number estimation, the regression-tree partition is performed in an allele-specific manner and at each region defined by the total copy number partition. The results are pruned back using the same cross-validation approach as was carried out for the total copy number estimation.

## Authors' contributions

JH developed and implemented the algorithm; all codes are written in R version 2.0.1.

WW, JC, JZ, RM, KJ, and MS were involved in the array data generation and independent verification using PCR molecular biology approaches. GL and XD were involved in bioinformatics analysis. SI and HA contributed to the creation of the algorithm framework. JH and MS wrote the manuscript and all authors read and approved the final manuscript.

## References

[B1] Sebat J, Lakshmi B, Troge J, Alexander J, Young J, Lundin P, Maner S, Massa H, Walker M, Chi M, Navin N, Lucito R, Healy J, Hicks J, Ye K, Reiner A, Gilliam TC, Trask B, Patterson N, Zetterberg A, Wigler M (2004). Large-scale copy number polymorphism in the human genome. Science.

[B2] Iafrate AJ, Feuk L, Rivera MN, Listewnik ML, Donahoe PK, Qi Y, Scherer SW, Lee C (2004). Detection of large-scale variation in the human genome. Nat Genet.

[B3] Sharp AJ, Locke DP, McGrath SD, Cheng Z, Bailey JA, Vallente RU, Pertz LM, Clark RA, Schwartz S, Segraves R, Oseroff VV, Albertson DG, Pinkel D, Eichler EE (2005). Segmental duplications and copy-number variation in the human genome. Am J Hum Genet.

[B4] Tuzun E, Sharp AJ, Bailey JA, Kaul R, Morrison VA, Pertz LM, Haugen E, Hayden H, Albertson D, Pinkel D, Olson MV, Eichler EE (2005). Fine-scale structural variation of the human genome. Nat Genet.

[B5] Feuk L, Carson AR, Scherer SW (2006). Structural variation in the human genome. Nat Rev Genet.

[B6] Conrad DF, Andrews TD, Carter NP, Hurles ME, Pritchard JK (2006). A high-resolution survey of deletion polymorphism in the human genome. Nat Genet.

[B7] Hinds DA, Kloek AP, Jen M, Chen X, Frazer KA (2006). Common deletions and SNPs are in linkage disequilibrium in the human genome. Nat Genet.

[B8] McCarrol SA, Hadnott TN, Perry GH, Sabeti PC, Zody MC, Barrett JC, Dallaire S, Gabriel SB, Lee C, Daly MJ, Altshuler DM (2006). Common deletion polymorphisms in the human genome. Nat Genet.

[B9] Pollack JR, Sorlie T, Perou CM, Rees CA, Jeffrey SS, Lonning PE, Tibshirani R, Botstein D, Borresen-Dale AL, Brown PO (2002). Microarray analysis reveals a major direct role of DNA copy number alteration in the transcriptional program of human breast tumors. Proc Natl Acad Sci U S A.

[B10] Pollack JR, Perou CM, Alizadeh AA, Eisen MB, Pergamenschikov A, Williams CF, Jeffrey SS, Botstein D, Brown PO (1999). Genome-wide analysis of DNA copy-number changes using cDNA microarrays. Nat Genet.

[B11] Snijders AM, Nowak N, Segraves R, Blackwood S, Brown N, Conroy J, Hamilton G, Hindle AK, Huey B, Kimura K, Law S, Myambo K, Palmer J, Ylstra B, Yue JP, Gray JW, Jain AN, Pinkel D, Albertson DG (2001). Assembly of microarrays for genome-wide measurement of DNA copy number. Nat Genet.

[B12] Pinkel D, Segraves R, Sudar D, Clark S, Poole I, Kowbel D, Collins C, Kuo WL, Chen C, Zhai Y, Dairkee SH, Ljung BM, Gray JW, Albertson DG (1998). High resolution analysis of DNA copy number variation using comparative genomic hybridization to microarrays. Nat Genet.

[B13] Lucito R, Healy J, Alexander J, Reiner A, Esposito D, Chi M, Rodgers L, Brady A, Sebat J, Troge J, West JA, Rostan S, Nguyen KC, Powers S, Ye KQ, Olshen A, Venkatraman E, Norton L, Wigler M (2003). Representational Oligonucleotide Microarray Analysis: A High-Resolution Method to Detect Genome Copy Number Variation. Genome Res.

[B14] Barrett MT, Scheffer A, Ben-Dor A, Sampas N, Lipson D, Kincaid R, Tsang P, Curry B, Baird K, Meltzer PS, Yakhini Z, Bruhn L, Laderman S (2004). Comparative genomic hybridization using oligonucleotide microarrays and total genomic DNA. Proc Natl Acad Sci U S A.

[B15] Brennan C, Zhang Y, Leo C, Feng B, Cauwels C, Aguirre AJ, Kim M, Protopopov A, Chin L (2004). High-resolution global profiling of genomic alterations with long oligonucleotide microarray. Cancer Res.

[B16] Hayashizaki Y, Hirotsune S, Okazaki Y, Hatada I, Shibata H, Kawai J, Hirose K, Watanabe S, Fushiki S, Wada S, Sugimoto T, Kobayakawa K, Kawara T, Katsuki M, Shibuya T, Mukai T (1993). Restriction landmark genomic scanning method and its various applications. Electrophoresis.

[B17] Schrock E, du Manoir S, Veldman T, Schoell B, Wienberg J, Ferguson-Smith MA, Ning Y, Ledbetter DH, Bar-Am I, Soenksen D, Garini Y, Ried T (1996). Multicolor spectral karyotyping of human chromosomes. Science.

[B18] Lisitsyn N, Wigler M (1993). Cloning the differences between two complex genomes. Science.

[B19] Wang TL, Maierhofer C, Speicher MR, Lengauer C, Vogelstein B, Kinzler KW, Velculescu VE (2002). Digital karyotyping. Proc Natl Acad Sci U S A.

[B20] Tengs T, LaFramboise T, Den RB, Hayes DN, Zhang J, DebRoy S, Gentleman RC, O'Neill K, Birren B, Meyerson M (2004). Genomic representations using concatenates of Type IIB restriction endonuclease digestion fragments. Nucleic Acids Res.

[B21] Volik S, Zhao S, Chin K, Brebner JH, Herndon DR, Tao Q, Kowbel D, Huang G, Lapuk A, Kuo WL, Magrane G, De Jong P, Gray JW, Collins C (2003). End-sequence profiling: sequence-based analysis of aberrant genomes. Proc Natl Acad Sci U S A.

[B22] Pinkel D, Landegent J, Collins C, Fuscoe J, Segraves R, Lucas J, Gray J (1988). Fluorescence in situ hybridization with human chromosome-specific libraries: detection of trisomy 21 and translocations of chromosome 4. Proc Natl Acad Sci U S A.

[B23] Fodor SP, Rava RP, Huang XC, Pease AC, Holmes CP, Adams CL (1993). Multiplexed biochemical assays with biological chips. Nature.

[B24] Fodor SP, Read JL, Pirrung MC, Stryer L, Lu AT, Solas D (1991). Light-directed, spatially addressable parallel chemical synthesis. Science.

[B25] Pease AC, Solas D, Sullivan EJ, Cronin MT, Holmes CP, Fodor SP (1994). Light-generated oligonucleotide arrays for rapid DNA sequence analysis. Proc Natl Acad Sci U S A.

[B26] Lindblad-Toh K, Tanenbaum DM, Daly MJ, Winchester E, Lui WO, Villapakkam A, Stanton SE, Larsson C, Hudson TJ, Johnson BE, Lander ES, Meyerson M (2000). Loss-of-heterozygosity analysis of small-cell lung carcinomas using single-nucleotide polymorphism arrays. Nat Biotechnol.

[B27] Wang ZC, Lin M, Wei LJ, Li C, Miron A, Lodeiro G, Harris L, Ramaswamy S, Tanenbaum DM, Meyerson M, Iglehart JD, Richardson A (2004). Loss of heterozygosity and its correlation with expression profiles in subclasses of invasive breast cancers. Cancer Res.

[B28] Mei R, Galipeau PC, Prass C, Berno A, Ghandour G, Patil N, Wolff RK, Chee MS, Reid BJ, Lockhart DJ (2000). Genome-wide detection of allelic imbalance using human SNPs and high-density DNA arrays. Genome Res.

[B29] Kennedy GC, Matsuzaki H, Dong S, Liu WM, Huang J, Liu G, Su X, Cao M, Chen W, Zhang J, Liu W, Yang G, Di X, Ryder T, He Z, Surti U, Phillips MS, Boyce-Jacino MT, Fodor SP, Jones KW (2003). Large-scale genotyping of complex DNA. Nat Biotechnol.

[B30] Matsuzaki H, Loi H, Dong S, Tsai YY, Fang J, Law J, Di X, Liu WM, Yang G, Liu G, Huang J, Kennedy GC, Ryder TB, Marcus GA, Walsh PS, Shriver MD, Puck JM, Jones KW, Mei R (2004). Parallel genotyping of over 10,000 SNPs using a one-primer assay on a high density oligonucleotide array. Genome Res.

[B31] Janne PA, Li C, Zhao X, Girard L, Chen TH, Minna J, Christiani DC, Johnson BE, Meyerson M (2004). High-resolution single-nucleotide polymorphism array and clustering analysis of loss of heterozygosity in human lung cancer cell lines. Oncogene.

[B32] Irving JA, Bloodworth L, Bown NP, Case MC, Hogarth LA, Hall AG (2005). Loss of heterozygosity in childhood acute lymphoblastic leukemia detected by genome-wide microarray single nucleotide polymorphism analysis. Cancer Res.

[B33] Hu N, Wang C, Hu Y, Yang HH, Giffen C, Tang ZZ, Han XY, Goldstein AM, Emmert-Buck MR, Buetow KH, Taylor PR, Lee MP (2005). Genome-wide association study in esophageal cancer using GeneChip mapping 10K array. Cancer Res.

[B34] Bignell GR, Huang J, Greshock J, Watt S, Butler A, West S, Grigorova M, Jones KW, Wei W, Stratton MR, Futreal PA, Weber B, Shapero MH, Wooster R (2004). High-resolution analysis of DNA copy number using oligonucleotide microarrays. Genome Res.

[B35] Huang J, Wei W, Zhang J, Liu G, Bignell GR, Stratton MR, Futreal PA, Wooster R, Jones KW, Shapero MH (2004). Whole genome DNA copy number changes identified by high density oligonucleotide arrays. Hum Genomics.

[B36] Rauch A, Ruschendorf F, Huang J, Trautmann U, Becker C, Thiel C, Jones KW, Reis A, Nurnberg P (2004). Molecular karyotyping using an SNP array for genomewide genotyping. J Med Genet.

[B37] Zhou X, Mok SC, Chen Z, Li Y, Wong DT (2004). Concurrent analysis of loss of heterozygosity (LOH) and copy number abnormality (CNA) for oral premalignancy progression using the Affymetrix 10K SNP mapping array. Hum Genet.

[B38] Raghavan M, Lillington DM, Skoulakis S, Debernardi S, Chaplin T, Foot NJ, Lister TA, Young BD (2005). Genome-wide single nucleotide polymorphism analysis reveals frequent partial uniparental disomy due to somatic recombination in acute myeloid leukemias. Cancer Res.

[B39] Wong KK, Tsang YT, Shen J, Cheng RS, Chang YM, Man TK, Lau CC (2004). Allelic imbalance analysis by high-density single-nucleotide polymorphic allele (SNP) array with whole genome amplified DNA. Nucleic Acids Res.

[B40] Herr A, Grutzmann R, Matthaei A, Artelt J, Schrock E, Rump A, Pilarsky C (2005). High-resolution analysis of chromosomal imbalances using the Affymetrix 10K SNP genotyping chip. Genomics.

[B41] Speicher MR, Carter NP (2005). The new cytogenetics: blurring the boundaries with molecular biology. Nat Rev Genet.

[B42] Matsuzaki H, Dong S, Loi H, Di X, Liu G, Hubbell E, Law J, Berntsen T, Chadha M, Hui H, Yang G, Kennedy GC, Webster TA, Cawley S, Walsh PS, Jones KW, Fodor SPA, Mei R (2004). Genotyping over 100,000 SNPs on a pair of oligonucleotide arrays. Nat Methods.

[B43] Klein RJ, Zeiss C, Chew EY, Tsai JY, Sackler RS, Haynes C, Henning AK, Sangiovanni JP, Mane SM, Mayne ST, Bracken MB, Ferris FL, Ott J, Barnstable C, Hoh J (2005). Complement factor H polymorphism in age-related macular degeneration. Science.

[B44] Slater HR, Bailey DK, Ren H, Cao M, Bell K, Nasioulas S, Henke R, Choo KH, Kennedy GC (2005). High-Resolution Identification of Chromosomal Abnormalities Using Oligonucleotide Arrays Containing 116,204 SNPs. Am J Hum Genet.

[B45] Breiman L, Friedman J, Olshen R, Stone C (1984). Classification and Regression Trees.

[B46] Lin M, Wei LJ, Sellers WR, Lieberfarb M, Wong WH, Li C (2004). dChipSNP: significance curve and clustering of SNP-array-based loss-of-heterozygosity data. Bioinformatics.

[B47] Nannya Y, Sanada M, Nakazaki K, Hosoya N, Wang L, Hangaishi A, Kurokawa M, Chiba S, Bailey DK, Kennedy GC, Ogawa S (2005). A robust algorithm for copy number detection using high-density oligonucleotide single nucleotide polymorphism genotyping arrays. Cancer Res.

[B48] Slamon DJ, Godolphin W, Jones LA, Holt JA, Wong SG, Keith DE, Levin WJ, Stuart SG, Udove J, Ullrich A, Press MF (1989). Studies of the HER-2/neu proto-oncogene in human breast and ovarian cancer. Science.

[B49] Kallioniemi A, Kallioniemi OP, Piper J, Tanner M, Stokke T, Chen L, Smith HS, Pinkel D, Gray JW, Waldman FM (1994). Detection and mapping of amplified DNA sequences in breast cancer by comparative genomic hybridization. Proc Natl Acad Sci U S A.

[B50] Kallioniemi OP, Kallioniemi A, Kurisu W, Thor A, Chen LC, Smith HS, Waldman FM, Pinkel D, Gray JW (1992). ERBB2 amplification in breast cancer analyzed by fluorescence in situ hybridization. Proc Natl Acad Sci U S A.

[B51] Hastie T, Tibshirani R, Friedman J (2001). The Elements of Statistical Learning, Data Mining, Inference, and Prediction.

[B52] Lai WR, Johnson MD, Kucherlapati R, Park PJ (2005). Comparative analysis of algorithms for identifying amplifications and deletions in array CGH data. Bioinformatics.

[B53] Laframboise T, Weir BA, Zhao X, Beroukhim R, Li C, Harrington D, Sellers WR, Meyerson M (2005). Allele-specific amplification in cancer revealed by SNP array analysis. PLoS Comput Biol.

[B54] Fridlyand J, Snijders AM, Pinkel D, Albertson DG, Jain AN (2004). Hidden Markov models approach to the analysis of array CGH data. Journal of Multivariate Analysis.

[B55] Zhao X, Weir BA, LaFramboise T, Lin M, Beroukhim R, Garraway L, Beheshti J, Lee JC, Naoki K, Richards WG, Sugarbaker D, Chen F, Rubin MA, Janne PA, Girard L, Minna J, Christiani D, Li C, Sellers WR, Meyerson M (2005). Homozygous deletions and chromosome amplifications in human lung carcinomas revealed by single nucleotide polymorphism array analysis. Cancer Res.

[B56] Olshen AB, Venkatraman ES, Lucito R, Wigler M (2004). Circular binary segmentation for the analysis of array-based DNA copy number data. Biostatistics.

[B57] Olshen AB, Venkatraman ES (2002). Change-point analysis of array-based comparative genomic hybridization data.

[B58] Daruwala RS, Rudra A, Ostrer H, Lucito R, Wigler M, Mishra B (2004). A versatile statistical analysis algorithm to detect genome copy number variation. Proc Natl Acad Sci U S A.

[B59] Wang P, Kim Y, Pollack J, Narasimhan B, Tibshirani R (2005). A method for calling gains and losses in array CGH data. Biostatistics.

[B60] Di X, Matsuzaki H, Webster TA, Hubbell E, Liu G, Dong S, Bartell D, Huang J, Chiles R, Yang G, Shen MM, Kulp D, Kennedy GC, Mei R, Jones KW, Cawley S (2005). Dynamic model based algorithms for screening and genotyping over 100K SNPs on oligonucleotide microarrays. Bioinformatics.

[B61] Copy Number Analyzer for GeneChip. http://www.genome.umin.jp.

[B62] dChip Software: Gene Expression Microarray and SNP Microarray Analysis. http://www.dchip.org.

[B63] Collins FS, Brooks LD, Chakravarti A (1998). A DNA polymorphism discovery resource for research on human genetic variation. Genome Res.

[B64] Ishikawa S, Komura D, Tsuji S, Nishimura K, Yamamoto S, Panda B, Huang J, Fukayama M, Jones KW, Aburatani H (2005). Allelic dosage analysis with genotyping microarrays. Biochem Biophys Res Comm.

